# APOE4 Exacerbates Cerebral Tau Pathology Through Cholesterol‐Induced Degradation of Phosphatase in Atherosclerosis

**DOI:** 10.1111/cns.70536

**Published:** 2025-07-30

**Authors:** Jiuyang Ding, Baofei Sun, Yang Gao, Cihang Gu, Jian Zhang, Li Wang, Jie Zheng, Bing Xia, Xiaolan Qi

**Affiliations:** ^1^ School of Forensic Medicine Guizhou Medical University Guiyang China; ^2^ Key Laboratory of Human Brain Bank for Functions and Diseases of Department of Education of Guizhou Province Guizhou Medical University Guiyang China; ^3^ Key Laboratory of Endemic and Ethnic Diseases, Ministry of Education Guizhou Medical University Guiyang China; ^4^ Department of Radiology Zhongnan Hospital of Wuhan University Wuhan China; ^5^ Guangzhou Key Laboratory of Forensic Multi‐Omics for Precision Identification, School of Forensic Medicine Southern Medical University Guangzhou China; ^6^ Neuroscience Research Institute and Department of Neurobiology, School of Basic Medical Sciences, Key Laboratory for Neuroscience, Ministry of Education/National Health Commission Peking University Beijing China

**Keywords:** Alzheimer's disease, APOE4, atherosclerosis, cholesterol, tau protein

## Abstract

**Background:**

Apolipoprotein epsilon4 allele (APOE4) is a common risk factor for atherosclerosis (AS) and neurodegenerative diseases like Alzheimer's disease (AD), but whether and how APOE4 induces AD‐like neuropathies in the brain of AS pathology remains poorly characterized.

**Methods:**

By combining postmortem AS human brains and APOE4 knock‐in mice, we examined the effects of APOE4 on tau and related neuropathological changes in the brain of AS. Behavioral and pathological observations were used to evaluate the protective effects of facilitating cholesterol transport in APOE4 AS mice.

**Results:**

Here, we showed that APOE4 carriers exhibited higher AD‐like phosphorylated Tau (p‐Tau) levels in AS postmortem brains. Knocking in human APOE4 in high fat diet‐fed mice induced AS pathology and coupled AS and AD. APOE4 promoted cerebral cholesterol content, which could trigger protein phosphatase 2A (PP2A) degradation. We further demonstrated cholesterol could facilitate the ubiquitination of PP2A B and PP2A C, which were regulatory and catalytic subunits of PP2A respectively, leading to PP2A B and PP2A C degradation through the ubiquitin‐proteasome system. Reduced PP2A B and PP2A C resulted in cerebral Tau hyperphosphorylated at multiple AD‐associated sites. The APOE4 AS mice exhibited an AD‐like phenotype, including synaptic degeneration, blood‐brain barrier breakdown, glial activation, and cognitive impairment simultaneously. Pharmacologically facilitating cholesterol transport alleviated neuropathologies in APOE4 AS mice.

**Conclusion:**

Altogether, these results suggested a role of the APOE4 in linking AS with Tau neuropathology, which might increase the risk of related neurodegenerative diseases for AS patients.

## Graphical abstract

Cholesterol couples atherosclerosis (AS) and Alzheimer (AD) ‐like tau pathology in APOE4 carriers. Promoting cholesterol homeostasis was effective in alleviating tau pathology and decreasing the risk for AD in AS patients of APOE4 genotype.

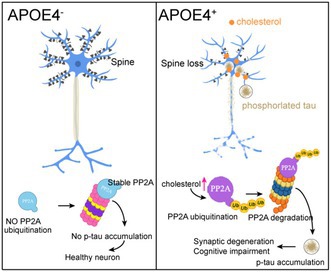

**Abbreviations**
AD: Alzheimer's disease
APOE4: apolipoprotein E ε4
AS: atherosclerosis
Aβ: amyloid‐β
CA1: cornu ammonis
CC: corpus callosum
COD: cause of death
Co‐IP: co‐immunoprecipitation
DG pl.: dendate gyrus polymorph layer
DG: dentate gyrus
EC: entorhinal cortex
FFPE: formalin‐fixed and paraffin‐embedded
HE: hematoxylin and eosin
HFD: high fat diet
HP‐β‐CD: 2‐hydroxypropyl‐β‐cyclodextrin
IHC: immunohistochemistry
MWM: Morris‐water maze
NOR: novel object recognition
NR2A: N‐methyl‐D‐aspartate receptor subunit A
NR2B: N‐methyl‐D‐aspartate receptor subunit B
PP2A: protein phosphatase
PSD95: post synaptic density protein 95
p‐Tau: phosphorylated tau
TEM: transmission electron microscopy

## 1 Introduction

Atherosclerosis (AS) is characterized by accumulation of lipids and formation of plaques in the arterial vessel wall, especially in coronary arteries and the aorta [1], as well as foam cell formation, vascular endothelial cell injury, etc. [2]. AS plaques narrow the arterial lumen and block blood flow, leading to acute cardiovascular events such as acute myocardial infarction, stroke, etc. [3]. It has been widely recognized that AS patients also show cognitive decline and have higher risk of dementia like Alzheimer's disease (AD) [4], the most common form of dementia in the elderly, which exhibits typical neuropathological features of amyloid‐β (Aβ) deposits, neurofibrillary tangles composed of hyperphosphorylated tau protein, and neuroinflammation [5]. However, pathophysiological factors linking the AS with AD remain elusive.

Genome‐wide association analysis has showed that the *E4* allele variant of the *APOE* gene (*APOE4*) was a common risk factor for AS and AD [6, 7]. APOE4 has been implicated in the pathogenesis of atherosclerosis by contributing to dysregulation of intracellular cholesterol balance, promoting inflammation within the arterial wall, and potentially exacerbating metabolic dysfunction, oxidative stress, and blood–brain barrier disruption. In contrast, another variant of the APOE gene, APOE2, is associated with lower LDL cholesterol levels and may confer a protective effect against atherosclerosis [8]. Meanwhile, individuals carrying the *APOE4* allele also have a three‐to‐eight fold increase in risk for developing AD [9], presumedly through exacerbating amyloid‐β deposition and p‐Tau aggregation [10, 11, 12]. These findings suggest a linking role of APOE4 between AS and AD.

The APOE4 polymorphism interfered with its ability of lipid transport [7], which promotes lipid droplets accumulation and dysregulation of cholesterol homeostasis in the brain, thus aggravating neuropathology in AD mice [13]. Especially, APOE4 could exacerbate tau hyperphosphorylation in mice [5], while the underlying mechanism remains to be elucidated. Phosphorylation of tau in AD can be at least partly attributed to the loss‐of‐function of phosphatases like protein phosphatase 2A (PP2A) [14]. However, whether and how APOE4 affects PP2A in AS is not known.

Herein, we sought to measure tau‐related neuropathology in AS brain of APOE4 carriers and noncarriers, respectively, and further investigate how APOE4 affects p‐Tau accumulation through cholesterol‐induced dysregulation of PP2A.

## 2 Methods

### 2.1 Human Brain Samples

A total of 25 human brain hippocampal tissues and coronary arteries were obtained from the School of Forensic Medicine of Guizhou Medical University. Clinical information including gender, age, postmortem interval (PMI), comorbidity, and cause of death (COD) was listed in the Table S1. Hippocampal specimens were dissected from the same level along the coronary plane during autopsy. Tissues were stored in liquid nitrogen or fixed in 4% PFA for immunoblot and/or histological analysis, respectively. All experiments were approved by the Ethics Committee of Guizhou Medical University (Approval number: 2023‐91). Written informed consent was obtained from family members of all deceased.

### 2.2 Animals

APOE ε3/ε3 and APOE ε4/ε4 mice (hereafter called APOE3 and APOE4 mice, respectively, male:female = 1:1) were purchased from Jackson Laboratory. Nine‐month‐old APOE3 and APOE4 mice were fed a high fat diet (HFD) for 4 months to mimic AS in humans and were sacrificed at 13 months old after behavioral testing. 2‐hydroxypropyl‐β‐cyclodextrin (HP‐β‐CD, Cat# H107, Sigma‐Aldrich, dissolved in saline, 2 g/kg body weight, subcutaneously) was treated for 8 weeks from the age of 11–13 months for the therapy. All animal experiments were conducted in accordance with National Institutes of Health (NIH) guidance and were approved by the Guizhou Medical University Animal Care and Use Committee.

### 2.3 Histological Staining and Immunohistochemistry (IHC)

Coronary arteries and brain tissues of humans or mice were fixed in 4% PFA, dehydrated, and embedded in paraffin. Three‐μm sections of formalin‐fixed and paraffin‐embedded (FFPE) tissues were obtained by microtome (RM2235, Leica, Germany). Sections were dewaxed and rehydrated. For hematoxylin and eosin (HE) staining, sections were stained with hematoxylin for 20 s before rinsing in eosin for 1 min. For IHC, sections were rinsed in 0.01 M sodium citrate for antigen retrieval before blocking in 3% hydrogen peroxide for 10 min, and subsequently incubated with primary antibodies (summarized in Table S2) at 4°C overnight, and with secondary antibodies for 90 min at room temperature. Reactions were developed with a streptavidin‐HRP DAB kit (Cat#CW2069, CW bio, China). Images were captured using a microscope (CX23, Olympus, Japan). For quantification, one average was plotted for 4–6 images taken across the sections using ImageJ software (NIH). All data were analyzed by individuals blinded to the experimental groupings.

### 2.4 Western Blotting and Co‐Immunoprecipitation (Co‐IP)

Brain homogenates or cultured cells were lysed in RIPA buffer. Protein quantification was performed using a BCA protein assay kit (Cat#P0009, Beyotime, China) according to the manufacturer's protocol. Samples were denatured at 95°C for 10 min and separated by SDS‐PAGE. Proteins were transferred to PVDF membranes, which were then incubated with primary antibodies at 4°C overnight. Primary antibodies were summarized in Table S2. After incubating with the IgG HRP‐linked secondary antibodies, the membranes were developed for reactions with ECL reagents (Cat#WBKLS0500, Merk, USA). The band intensities were quantified by ImageJ (NIH).

For Co‐IP test, the tissues or cells were lysed using RIPA buffer. The Co‐IP kit (Cat#abs955, absin, China) was used according to the manufacturer's protocol. Briefly, 5 μL Protein A + G beads were added into the protein lysate (500 μg, 1 mg/mL). After shaking on a vibrator at 4°C for 0.5 h, the mixture was centrifuged. Primary antibodies including PP2A B and PP2A C (Table S2) were incubated with the supernatant at 4°C overnight. 5 μL Protein A + G beads were incubated with the supernatant at 4°C overnight. After 3 times of centrifugation at 12,000 *g* for 1 min, the deposit was collected, and the beads mixture was washed with 0.1 M PBS for 3 times, the deposit was mixed with 1× SDS, heated at 95°C for 1 min, and then centrifuged at 14,000 *g* for 1 min, the supernatant was used for immunoblotting.

### 2.5 Quantitative PCR (qPCR)

qPCR was conducted as in our previous studies [15]. Primers employed were as follows: PPP2R2C, F: 5′‐GATTACCGAACGAGACAAGAGG‐3′, R: 5′‐GAGATGGAGTTGATGTGGTAGG‐3′, PPP2R5B, F: 5′‐GCGGTATTTGGGACCCTCTA‐3′, R: 5′‐TTCTGCTGCTCCTGTTGTTTTT‐3′, PPP2R5D, F: 5′‐GCACACAGCAGTACAAGGC‐3′, R: 5′‐GGAGGAGCCCGAAACATAGG‐3′, PPP2R5E, F: 5′‐GACGGATTTTCTCGGAAGTCC‐3′, R: 5′‐GAGGTTGGAACGTC TTTCAGC‐3′. GAPDH, F: 5′‐ATGGGTGTGAACCACGAGA‐3′, R: CAGGGATG ATGTTCTGGGCA‐3′. mRNA levels of interested genes were normalized by GAPDH mRNA.

### 2.6 Golgi‐Cox Staining

Golgi‐cox staining was conducted using a Golgi staining kit (Cat#PK401, FD NeuroTechnologies, USA) as previously reported [16]. Briefly, mouse brains were dipped into the Golgi solution for 5 weeks in dark and then were transferred into the protection solution for 1 week at 4°C in the dark. The tissues were sectioned to 100 μm in thickness and then picked up onto slides. Sections were then rinsed in 50% ethanol for 5 min before being washed in PBS for 5 min. After being rinsed in 5% sodium thiosulfate in the dark for 10 min, the sections were dehydrated using ascending gradient ethanol. Images were taken using a microscope (SV120, Olympus, Tokyo).

### 2.7 Transmission Electron Microscopy (TEM)

As in our previous studies [17, 18], mice hippocampal tissues were acutely dissociated, and then fixed in 2.5% glutaraldehyde for 4 h before being rinsed in a mixture of 1% osmium tetroxide mixed and 1% potassium ferrocyanide for 1 h. After being dehydrated in an ascending concentration gradient of ethanol, samples were incubated in Epon resin at 65°C for 2 days. Sections of 5 nm in thickness were cut using an ultramicrotome, and then picked up onto copper grids. The specimen was stained with lead citrate. Images were taken using an electron microscope (Tecnai G2 F20, FEI, USA) with a 4 k CCD camera.

### 2.8 Serum Cholesterol Analysis

The measurement of serum cholesterol was performed using a cholesterol quantitation kit (ab65359, Abcam, USA) according to the manufacturer's protocols. Briefly, serum was acquired and then diluted 10‐fold in assay buffer II. Total lipids were extracted by resuspending the serum in 200 μL of chloroform: isopropanol: NP‐40 (7:11:0.1). Samples were centrifuged at 15,000 *g* for 10 min and dried. The free and total cholesterol was tested using free cholesterol reaction mix and total cholesterol reaction mix respectively. The cholesterol contents were calculated according to the standard curve.

### 2.9 Behavioral Tests

For Morris‐water maze (MWM) tests, a water tank (120 cm in diameter) was filled with fresh water (20 ~ 22°C, opacified with titanium dioxide). A hidden platform (10 cm in diameter) was located 1 cm below the water surface. Mice were placed into the tank from a random quadrant in the training session and allowed 60 s for free moving. The mice were picked to the platform if they did not find the platform within 60 s. The escape latency was recorded as the time the mice found the hidden platform. In the probe trial test, the platform was removed, and mice were allowed to swim for 60 s in the tank. The number of mice crossing through the quadrant where the platform was previously located was recorded.

For the novel object recognition (NOR) test, in the training phase, mice were placed in the box containing two identical objects (wooden balls), and in the test phase, mice were placed into the same box, but one object was replaced with a novel object (cubic wooden object) at the same location. Each phase lasts for 10 min. The discrimination index (DI) = (time spent exploring novel object—time spent exploring familiar object)/total time spent exploring these two objects × 100%. The exploring action was considered if mice were nose touching or within 2 cm of the object.

### 2.10 Primary Cell Culture and Immunofluorescence Staining

Primary hippocampal neuron culturing was performed as in our previous studies [19, 20]. Briefly, hippocampal tissues of APOE3 and APOE4 mice at E12 ~ 13 days were dissociated and digested. Neurobasal‐A (10,888,022, Gibico, Invitrogen) and B27 (175044‐044, Gibico, Invitrogen) were used to culture the neurons in 6 or 12 wells. After 1 week, the PBS or cholesterol (50 μg/mL, Cat#C4951, Sigma‐Aldrich, USA) was added into the medium, and washed out 1 h later. After washing with 0.1 M PBS, cells were fixed in 4% PFA at room temperature for 15 min, and then incubated with anti‐MAP2 antibody, and donkey anti‐rabbit secondary antibody Alexa Fluor 555 in turn (Table S2). Images were taken using a confocal microscope (LSM880, Zeiss, Germany).

### 2.11 Filipin Staining

Brain tissues were fixed in 4% PFA for 24 h. Sections (40 μm in thickness) were acquired using a microtome (CM1950, Leica, Germany), stained in filipin solution (0.25 mg/mL in PBS, Cat#F4767, Sigma‐Aldrich, USA) for 15 min, and then mounted onto glass. Images were taken using a confocal microscope (LSM880, Zeiss, Germany).

### 2.12 Statistical Analysis

All data were shown as mean ± SEM unless otherwise specified. All data distribution normality was determined by the Shapiro–Wilk test, and homogeneity of variance was verified using Levene's test. Statistical analysis was performed using SPSS 22.0 (IBM, New York, USA) and/or GraphPad Prism 8 (GraphPad, USA). Unpaired two‐tailed Student's *t*‐test or two‐way ANOVA followed by Bonferroni's post hoc test was performed for comparison. The *p* value < 0.05 was considered significant. Detailed statistical parameters including *n*, *T*, *p*, *r*, and *F* were listed in the figures, figure legends, or Table S3.

## 3 Results

### 3.1 Higher Degree of p‐Tau Accumulation and Related Pathologies in the Hippocampus of APOE4‐Carried AS Patients

**FIGURE 1:**
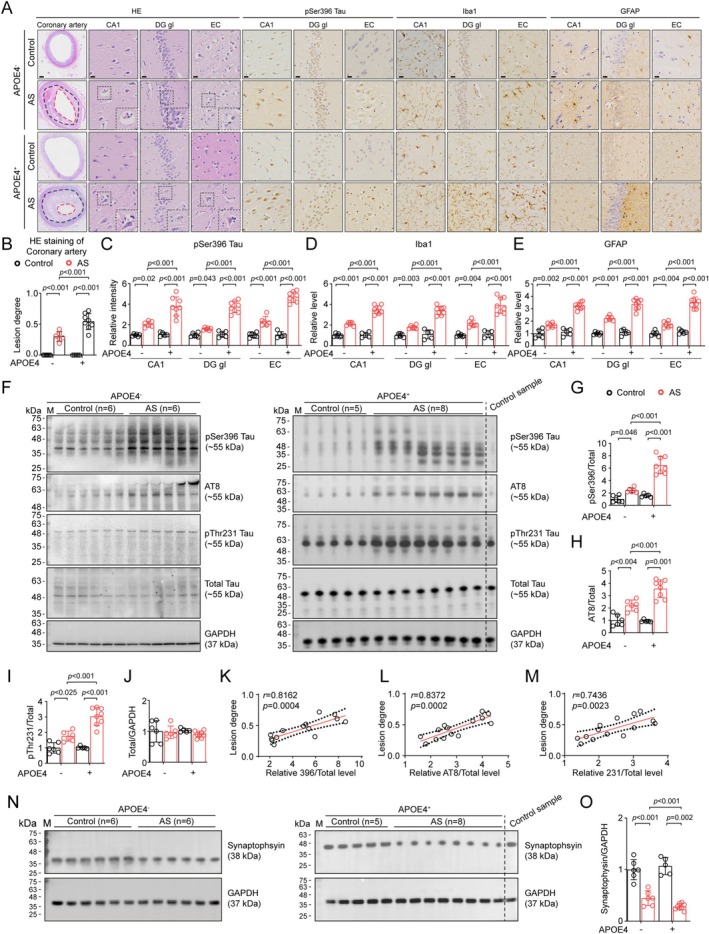
Higher degree of tau hyperphosphorylation and related neuropathologies in the hippocampus of APOE4‐carried AS patients. Representative HE or IHC images of coronary arteries (bar, 500 μm) and hippocampal tissues (bar, 25 μm), respectively, in postmortem human samples. For coronary arteries HE staining, the black dashed lines indicated original lumen of the ateries, and the red dashed lines indicated the narrowed lumen of the arteries. For hippocampal tissues HE staining, the magnified area in the lower right corner of each image showed neuronophagia. (B–E) APOE4^+^ AS patients had more prominent lesion degree in coronary arteries (B), and exacerbated accumulation of pSer396 tau (C), Iba1‐positive Microglia (D), and GFAP‐positive astrocytes in hippocampal CA1, dentate gyrus (DG) granular cell layer (gl) and entorhinal cortex (EC). Two‐way ANOVA followed by Bonferroni's post hoc test, *n* = 5 ~ 8 in each group. (F–J) APOE4^+^ AS patients had more prominent increase of p‐Tau at pSer396 (G), AT8 (H), pThr231 (I) but no change in total tau (J) in the hippocampus of AS patients. Two‐way ANOVA followed by Bonferroni's post hoc test, *n* = 5 ~ 8 in each group. (K–M) Pearson correlation analysis between p‐Tau levels and AS lesion degree in AS patients, *n* = 14 in total. The dashed lines represent 95% confidence intervals. (N, O) Immunoblot for Synaptophysin in human brain hippocampal tissues. (O) APOE4^+^ AS patients showed more prominent decrease of synaptophysin levels in the hippocampus. *n* = 5–8 in each group. Two‐way ANOVA followed by Bonferron's post hoc analysis.

We firstly measured the AS degree and hippocampal p‐Tau level in APOE4 carriers (APOE4^+^) and noncarriers (APOE4^−^) using postmortem human artery and brain samples, respectively. Plaques were visible in the coronary arterial lumina of all AS patients, but the stenosis degree was significantly higher in APOE4^+^ individuals (Figure 1A,B). Besides, we found in AS patients neuronophagia and neuropathologies at multiple brain areas including dentate gyrus (DG), CA1, and entorhinal cortex (EC) compared with healthy controls (Figure 1A, Figure S1A), and increase in the levels of p‐Tau (Ser396) accumulation as well as gliosis of microglia and astrocytes were all much marked in APOE4^+^ AS patients (Figure 1A, Figure S1A–D). The exacerbation of p‐Tau elevation in APOE4^+^ patients was observed at multiple AD‐associated epitopes including pSer396, AT8 (pSer202 & pThr205) and pThr231, even though total tau detected by Tau5 antibody remained unchanged (Figure 1F–J). Further analysis revealed that the levels of p‐Tau were positively correlated with the lesion degree of AS pathology (Figure 1K–M). In addition, we did not find any Aβ plaque in all human hippocampal samples (Figure S2A).

In consistent with the aggravation of p‐Tau accumulation in APOE4^+^ patients, we also observed a higher degree in the decrease of synaptic protein synaptophysin level (Figure 1N,O), which suggested exacerbated neurodegeneration induced by p‐Tau accumulation in APOE4^+^ patients.

### 3.2 More Potent Tau Hyperphosphorylation and Neurodegeneration in the Hippocampus of HFD‐Treated APOE4 Mice

**FIGURE 2:**
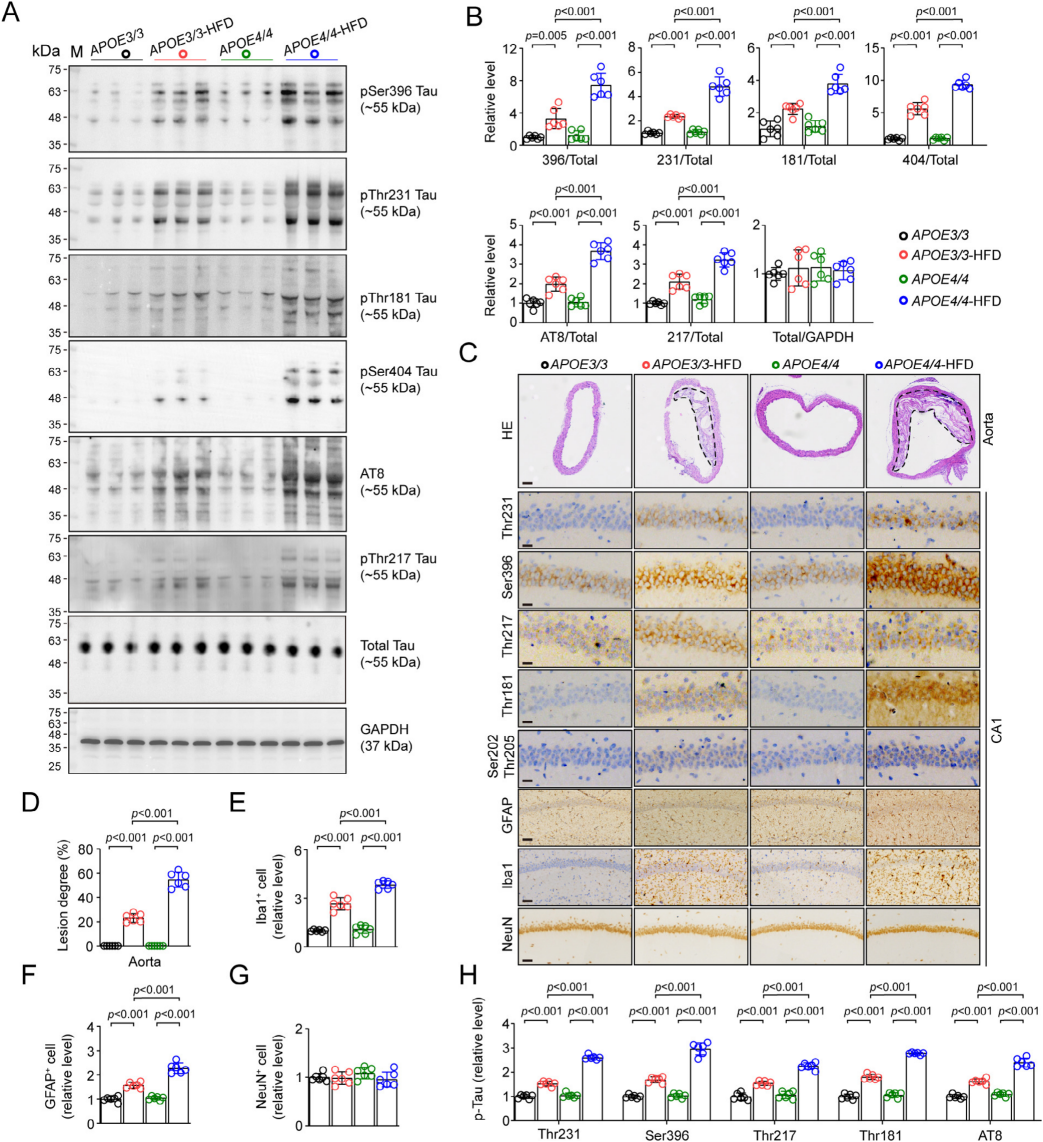
HFD‐treated APOE4 mice have higher degree of tau hyperphosphorylation in the hippocampus. (A, B) Western blot analysis showed more potent increase in p‐Tau at pSer396, Thr231, Thr181, pSer404, AT8 (pSer202/Thr205) and Thr217 but no significant change in total tau in HFD‐treated APOE4 mice. Data were normalized to total tau or GAPDH as indicated. *n* = 6 mice per group. Two‐way ANOVA followed by Bonferroni's post hoc tests. (C–H) HE or IHC staining exhibited exacerbated lesion degree in aortic arteries (D), with higher degree of gliosis (E, F), no change in NeuN‐positive neurons, and higher degree of tau hyperphosphorylation at variable epitopes (H). Bar was 100 μm for of aortic arteries images, and 25 μm for IHC images. *n* = 6 mice per group. Two‐way ANOVA followed by Bonferroni's post hoc tests.

We next examined the effects of APOE4 on p‐Tau and related pathologies in human APOE4 knock‐in mice fed with high fat diet (HFD), a model for simulating AS. APOE3 knock‐in mice were used as parallel controls. HFD significantly increased the level of p‐Tau at multiple sites, including Ser396, AT8, Thr231, Thr181, Thr217, and Ser404, with nonsignificant changes in total tau. Notably, similar to humans, the degree of p‐Tau increases in AS mice were much more potent in APOE4‐HFD mice compared with APOE3‐HFD (Figure 2A,B). Likewise, immunostaining also showed exacerbated AS phenotype in aortic arteries, as well as tau hyperphosphorylation and gliosis in APOE4‐HFD mice, even though the total number of NeuN‐positive neurons was comparable among all mice (Figure 2C–H). No Aβ plaque was observed in the brains of all these mice (Figure S2B).

Consistently, we also found aggravated microtubule depolymerization in APOE4‐HFD mice, a subcellular pathology tightly associated with tau hyperphosphorylation (Figure S3A,B).

**FIGURE 3:**
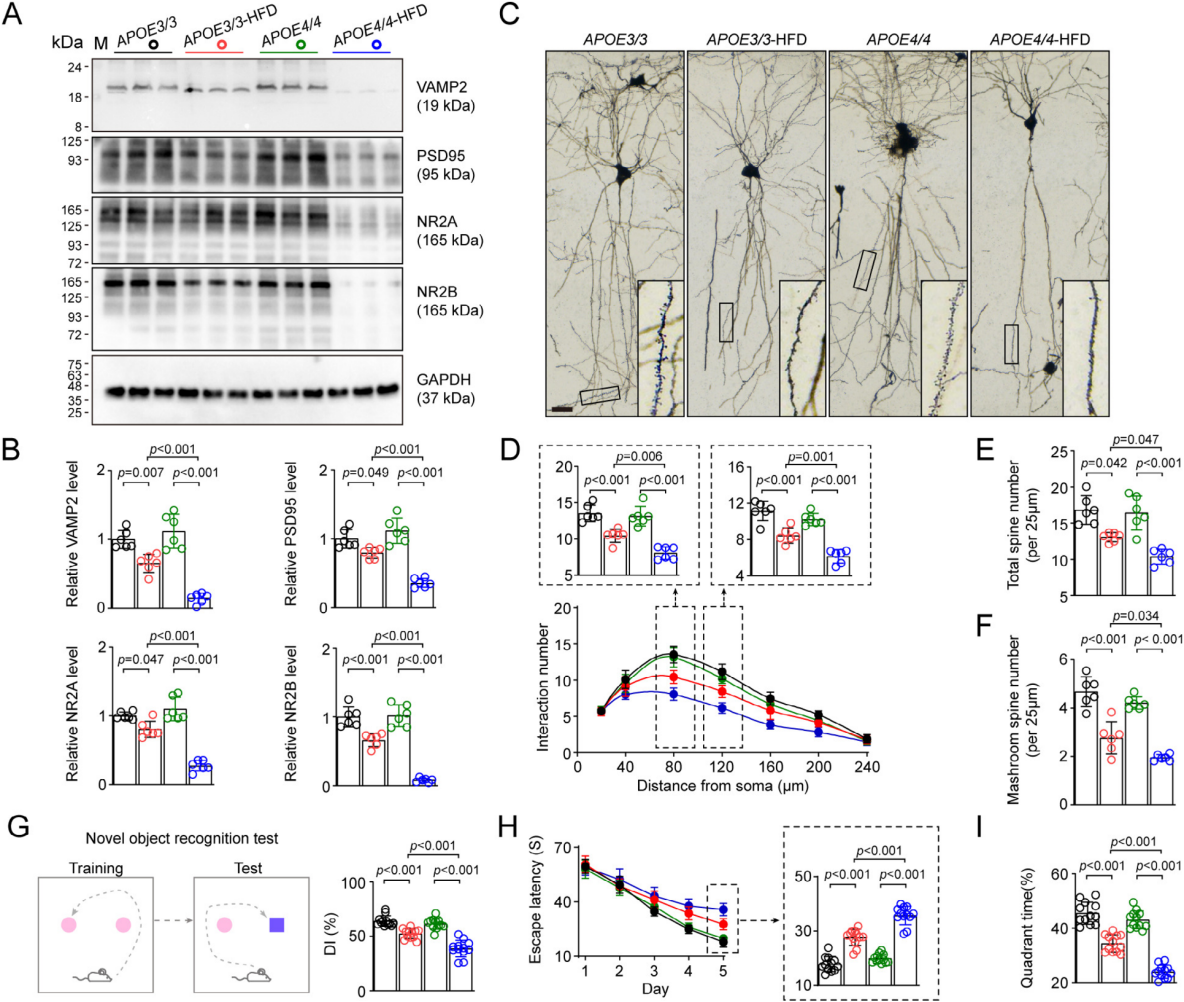
APOE4 aggravates synaptic degeneration in HFD‐treated APOE4 mice. (A, B) Western blotting indicates more potent decrease of synaptic proteins in hippocampal samples of HFD‐treated APOE4 mice. *n* = 6 mice per group. Two‐way ANOVA followed by Bonferroni's post hoc tests. (C) Representative images of CA1 pyramid neurons in Golgi staining. Bar, 25 μm. (D) Sholl analysis revealed more prominent decrease of dendrite complexity of CA1 pyramid neurons in HFD‐treated APOE4 mice. *n* = 6 mice per group. Two‐way ANOVA followed by Bonferroni's post hoc tests. (E, F) More potent decrease of total (E) and mushroom‐type (F) spine numbers of CA1 neurons in Golgi staining for HFD‐treated APOE4 mice. *n* = 6 mice per group. Two‐way ANOVA followed by Bonferroni's post hoc tests. (G) HFD‐treated APOE4 mice had lower discriminative index in the NOR test. *n* = 12 mice per group. Two‐way ANOVA followed by Bonferroni's post hoc tests. (H, I) HFD‐treated APOE4 mice had longer escape latency during the leaning phase (H) and less time in target quadrant in the test phase (I) of MWM test. *n* = 12 mice per group. Two‐way ANOVA followed by Bonferroni's post hoc tests.

Moreover, we observed aggravated decreases in synaptic protein levels, including VAMP2, PSD95, NR2A, and NR2B in APOE4‐HFD mice compared with APOE3‐HFD (Figure 3A,B). Golgi staining showed a more potent reduction in dendrite complexity and density of both total and mushroom‐type spines of hippocampal neurons in APOE4‐HFD mice (Figure 3C–F). These data suggested that APOE4 exacerbated the degeneration of hippocampal neurons in AS mice, which might be a consequence of abnormal p‐Tau accumulation [21].

Subsequently, we tested the effects of APOE4 on cognitive performances in AS mice. HFD treatment in both APOE3 and APOE4 mice resulted in a lower discriminative index in the NOR test, while the cognitive impairment was much more potent in APOE4 mice compared with APOE3 (Figure 3G,H). Similarly, in the learning phase of the MWM test, APOE4‐HFD mice had a much higher escape latency on day 5 than that of APOE3‐HFD mice, while in the test phase, APOE4‐HFD mice spent much less time in the target quadrant than APOE3‐HFD mice, indicating more exacerbated learning and memory impairments of APOE4 AS mice (Figure 3H,I).

### 3.3 APOE4‐Induced Cholesterol Accumulation Exacerbates Tau Hyperphosphorylation and Synaptic Degeneration

**FIGURE 4:**
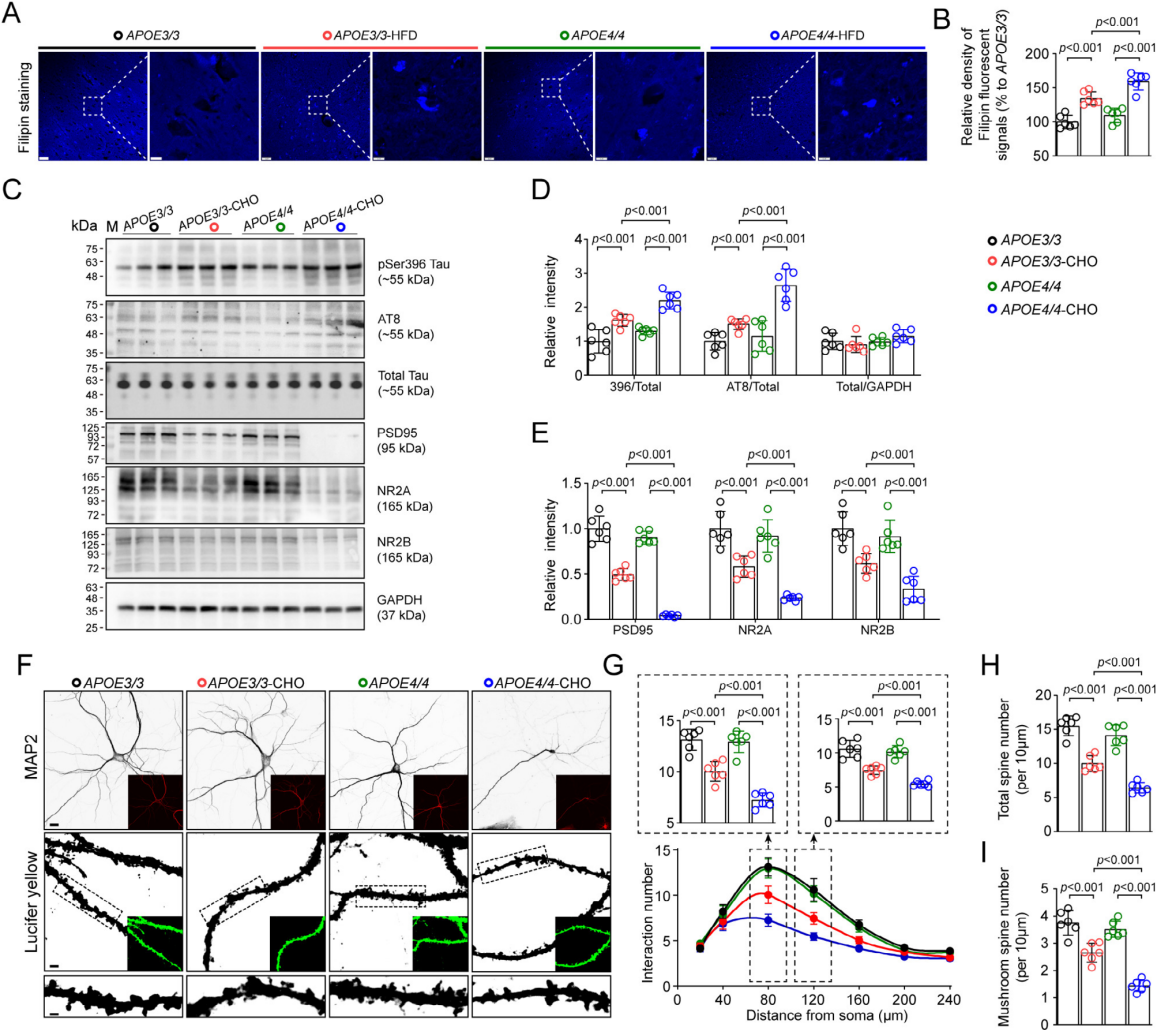
Cholesterol promotes tau hyperphosphorylation and synaptic degeneration in cultured hippocampal neurons. (A, B) Filipin staining exhibits higher level of cholesterol accumulation in the CA1 region of HFD‐APOE4 mice. Bar, 50 μm in low magnification images and 10 μm in the high magnification images. (C–E) Western blotting reveals more potent increase of p‐Tau and decrease of synaptic proteins in cholesterol‐treated APOE4 neurons. *n* = 6 wells of cells per group. Two‐way ANOVA followed by Bonferroni's post hoc tests. (F) Representative images of MAP2 IF staining (bar, 25 μm) and neurons with intracellular lucifer yellow dye injection (bar, 5 μm in upper panel and 2 μm in lower panel). (G) Sholl analysis finds more decrease in the dendrite complexity of cholesterol‐treated APOE4 neurons. Two‐way ANOVA followed by Bonferroni's post hoc tests. *n* = 6 wells of cells per group. (H) Cholesterol‐treated APOE4 neurons have decreased number of total (H) and mushroom‐type (I) dendrite spines. *n* = 6 wells of cell per group. Two‐way ANOVA followed by Bonferroni's post hoc tests.

Given that APOE4 has been widely recognized to be associated with cholesterol dysregulation in brain [22], we next tested the level of cholesterol in the serum and hippocampus of AS mice using ELISA and Filipin staining, respectively. HFD upregulated both the contents of free and total cholesterol in the serum of mice, but the increase of cholesterol was much higher in APOE4‐HFD mice compared with APOE3‐HFD (Figure S4). Likewise, the increase of cholesterol in the hippocampus was also much higher in APOE4‐HFD mice (Figure 4A,B). The aggravation of cholesterol accumulation in the brain of APOE4‐HFD mice might be associated with peripheral increase of cholesterol, since we found much more decrease in the number of tight junctions in the brain–blood barrier (BBB) in APOE4‐HFD mice compared with APOE3‐HFD (Figure S5A,B).

We next examined whether cholesterol accumulation is sufficient to induce tau hyperphosphorylation in primary cultured hippocampal neurons of APOE3 and APOE4 mice. Indeed, cholesterol treatment increased levels of p‐Tau at pSer396 and AT8 (pSer202 & pThr205) epitopes, as well as decreased levels of synaptic proteins including PAS95, NR2A, and NR2B, in which all changes were more potent in APOE4 neurons compared with APOE3 (Figure 4C–E). Moreover, we observed the morphologies of cultured neurons by MAP2 staining and intraneuronal lucifer yellow microinjection. Sholl analysis showed that cholesterol induced much more decrease in dendritic complexity of APOE4 neurons compared with APOE3. The cholesterol‐induced decreases in the number of both total and mushroom‐type spines also were more potent in APOE4‐CHO neurons compared with APOE3‐CHO neurons (Figure 4F–I).

### 3.4 APOE4 Exacerbates Tau Hyperphosphorylation Through Cholesterol‐Induced Enhancement of PP2A Proteolysis

**FIGURE 5:**
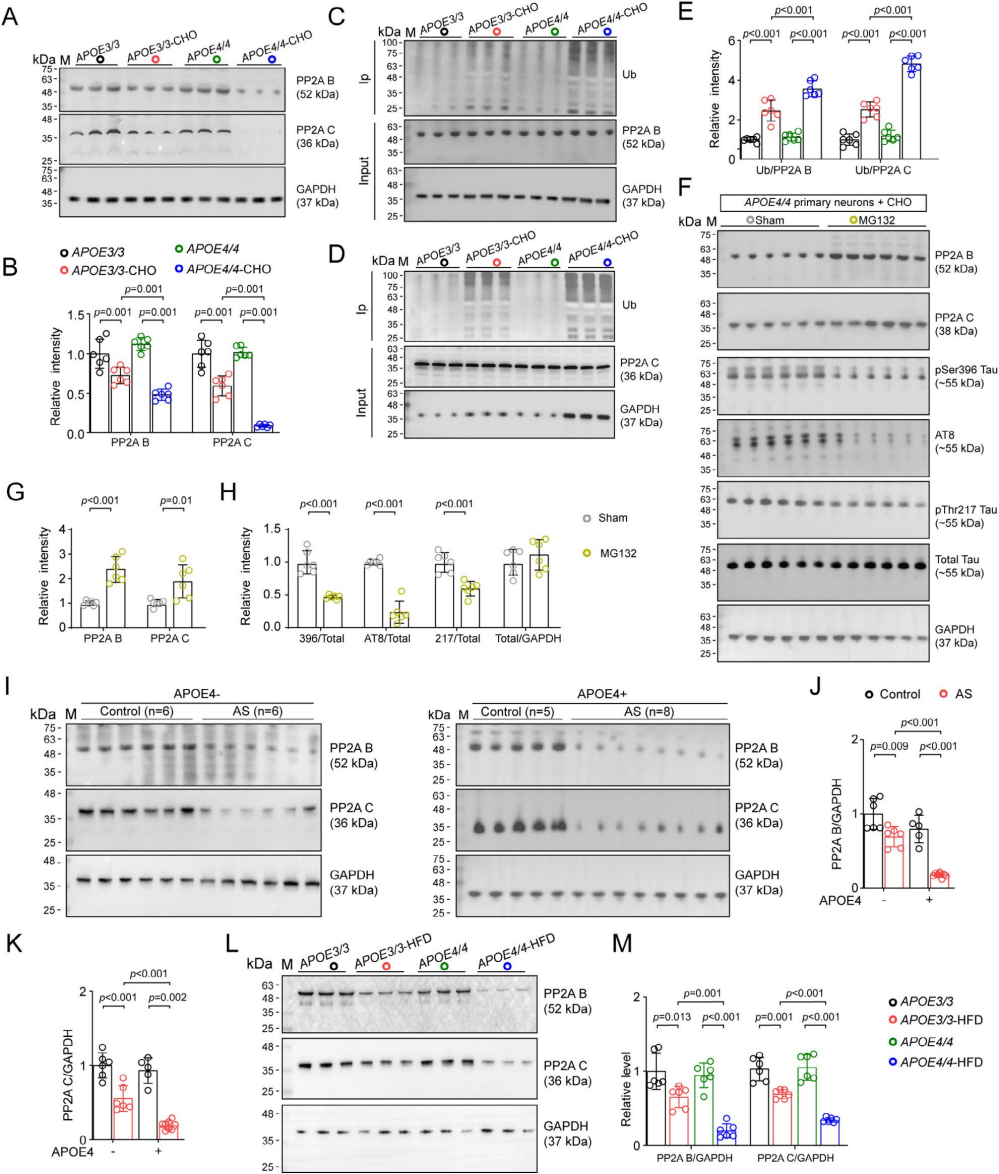
APOE4 exacerbates tau hyperphosphorylation through cholesterol‐induced enhancement of PP2A proteolysis. (A, B) Cholesterol‐treated APOE4 neurons had more potent decrease of PP2A B and C. *n* = 6 wells of cell per group. Two‐way ANOVA followed by Bonferroni's post hoc tests. (C, D) Co‐IP assay showed more potent upregulation of PP2A B and C ubiquitination level in cholesterol‐treated neurons. *n* = 6 wells of cell per group. Two‐way ANOVA followed by Bonferroni's post hoc tests. (F–H) MG132 increased the level of PP2A B and C (G), and decreased levels of p‐Tau in cholesterol‐treated APOE4 neurons. *n* = 6 wells of cell per group. Unpaired two‐tailed Student's *t*‐tests. (I–K) Exacerbated decrease of PP2A B and C in the hippocampus of APOE4^+^ AS patients. *n* = 6 person per group. Two‐way ANOVA followed by Bonferroni's post hoc tests. (L, M) Exacerbated decrease of PP2A B and C in HFD‐treated APOE4 mice. *n* = 6 mice per group, Two‐way ANOVA followed by Bonferroni's post hoc tests.

We aimed to investigate how tau phosphorylation was dysregulated by cholesterol accumulation. Since it has been reported that cholesterol was capable of regulating the activity of PP2A [23, 24], one of the most active tau phosphatases in the brain, we next examined changes in PP2A regulatory subunit B and catalytic subunit C in cultured hippocampal neurons treated with or without cholesterol. Inconsistent with changes in p‐Tau, we found that cholesterol induced decreases in protein levels of both PP2A B and C subunits, which were more potent in APOE4‐CHO neurons compared with APOE3‐CHO neurons (Figure 5A,B). By contrast, we did not find significant changes in PP2A B and C mRNAs among APOE3 and APOE4 neurons treated with or without cholesterol (Figure S6A,B), indicating that the accumulation of cholesterol induced a reduction of PP2A in a posttranslational manner.

In it has been recognized that cholesterol promoted p‐Tau aggregation through inhibiting ubiquitin‐mediated proteolysis [25, 26], therefore, we assessed how cholesterol determined the ubiquitination levels of PP2A B and C, and found that cholesterol treatment increased ubiquitination levels of both PP2A B and C subunits, which were more prominent in APOE4 neurons than APOE3 neurons (Figure 5C–E). Meanwhile, inhibition of proteasomal activity with MG132 prevented the cholesterol‐induced degradation of PP2A B and C, accompanied by a reduction of p‐Tau levels in APOE4 neurons (Figure 5F–H).

Consistently, we also observed lower PP2A B and C levels in the hippocampus of both APOE4^+^ AS patients (Figure 5I,J) and APOE4‐HFD mice (Figure 5L,M). Altogether, these findings suggested that cholesterol promoted p‐Tau accumulation in AS brains through enhancing the ubiquitin‐mediated PP2A proteolysis.

### 3.5 Aiding Cholesterol Homeostasis Alleviated Tau Pathology and Neurodegeneration in the Hippocampus of APOE4‐HFD Mice

**FIGURE 6:**
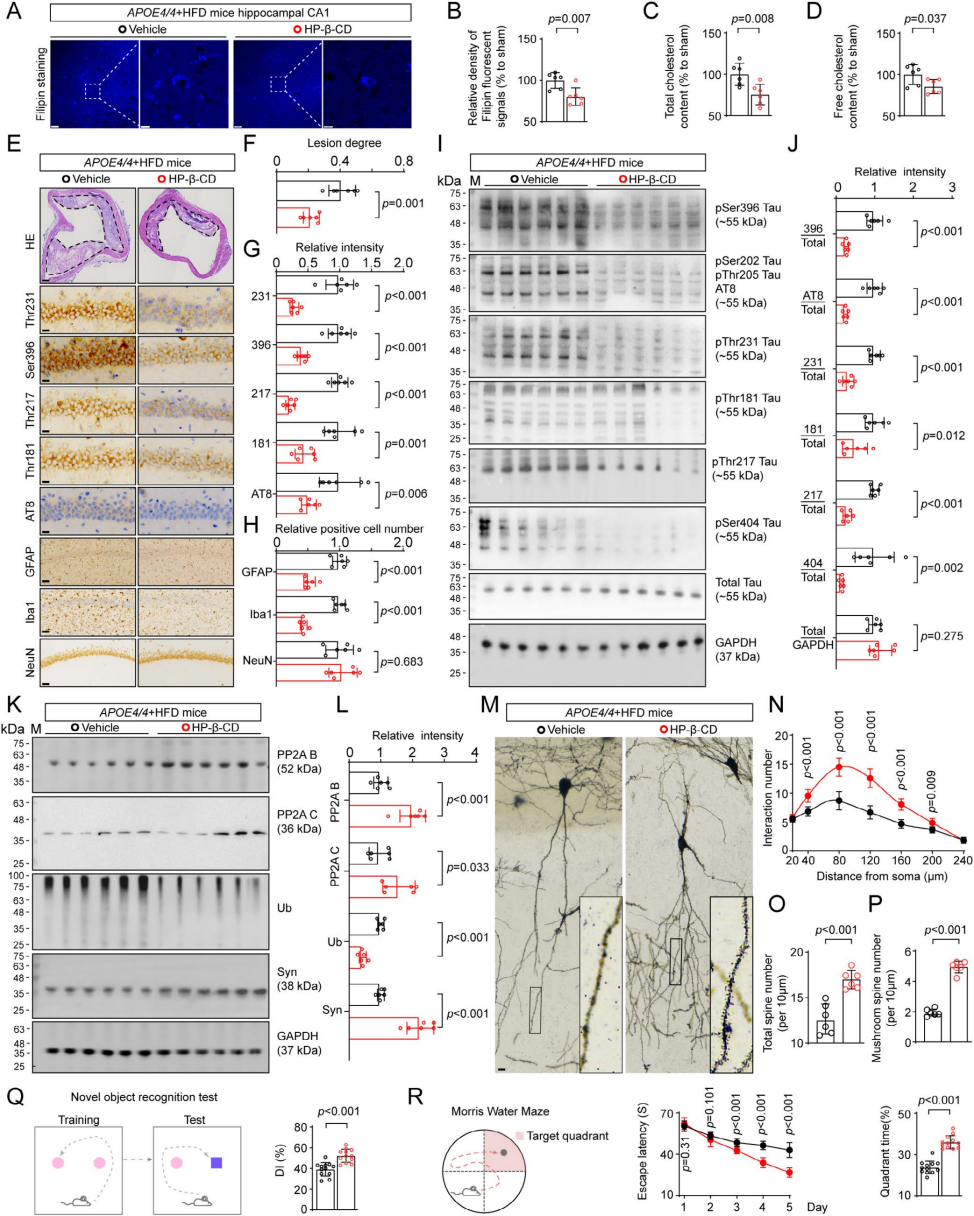
HP‐β‐CD alleviates tau pathology and improves cognition in APOE4 HFD mice. (A, B) Filipin staining showed lower cholesterol content in the CA1 region APOE4‐HFD mice treated with HP‐β‐CD. Bar, 50 μm in low magnification images and 10 μm in the high magnification images. *n* = 6 mice per group, unpaired two‐tailed Student's *t*‐tests. (C, D) HP‐β‐CD decreased total (C) and free (D) cholesterol content in the serum of APOE4‐HFD mice. *n* = 6 mice per group, unpaired two‐tailed Student's *t*‐tests. (E–H) HP‐β‐CD mitigated lesion degree of AS pathology (F), decreased levels of p‐Tau at multiple epitopes, and inhibited gliosis in the hippocampal CA1 of APOE4‐HFD mice. *n* = 6 mice per group, unpaired two‐tailed Student's *t*‐tests. (I, J) HP‐β‐CD decreased levels of p‐Tau in THE hippocampus of APOE4‐HFD mice. *n* = 6 mice per group, unpaired two‐tailed Student's *t*‐tests. (K, L) HP‐β‐CD increased levels of PP2A B and C, increased synaptophysin in the hippocampus of APOE4‐HFD mice. *n* = 6 mice per group, unpaired two‐tailed Student's *t*‐tests. (M–P) HP‐β‐CD increased dendrite complexity (N), and increased number of total or mushroom‐type spines of hippocampal CA1 pyramid neurons in APOE4‐HFD mice. *n* = 6 mice per group, unpaired two‐tailed Student's *t*‐tests. (Q) HP‐β‐CD increased discriminative index of APOE4‐HFD mice in NOR test. *n* = 12 mice per group, unpaired two‐tailed Student's *t*‐tests. (R) HP‐β‐CD improved learning and memory of APOE4‐HFD mice in MWM test. Repeated measures ANOVA followed by Bonferron's post hoc tests or unpaired two‐tailed Student's *t*‐tests. *n* = 12 mice per group.

Last but not least, we tested whether reduction of cholesterol content was effective in mitigating the p‐Tau and related neuropathology in AS mice. The 2‐hydroxypropyl‐β‐cyclodextrin (HP‐β‐CD), a molecule for manipulating cholesterol transport, was used to reduce cholesterol accumulation in vivo. Cholesterol content in the brain was reduced by 19.5% in HP‐β‐CD treated APOE4‐HFD mice compared with the vehicle group (Figure 6A,B). In serum, total and free cholesterol were reduced by 24.4% and 14.5%, respectively, after HP‐β‐CD treatment (Figure 6C,D). HP‐β‐CD administration significantly reduced the lesion area of the aortic arch in APOE4‐HFD mice, as well as downregulated gliosis and phosphorylation levels of tau at multiple sites including pSer396, AT8, pThr231, pThr181, pThr217, and pSer404, without showing significant impact on the total number of hippocampal neurons (Figure 6E–J). Consistently, HP‐β‐CD increased the protein levels of PP2A B and C, increased synaptophysin levels (Figure 6K,L), and improved neurite plasticity by increasing dendrite complexity and spine density (Figure 6M–P). Still, no Aβ plaque was observed in the brain of all these mice (Figure S2C).

Building on these findings, we tested whether aiding cholesterol homeostasis could alleviate memory impairments in APOE4‐HFD mice. Indeed, HP‐β‐CD significantly improved the learning and memory of APOE4‐HFD mice, as indicated by an increased discriminative index in NOR tests (Figure 6Q), and faster speed in learning to find the platform during the learning phase as well as more time spent in the target quadrant in the test phase in MWM tests (Figure 6R).

## 4 Discussion

By combining postmortem AS human brains and APOE4 knock‐in mice, we examined the effects of APOE4 on tau and related neuropathological changes in the brain of AS, and found that APOE4 promotes cholesterol accumulation, which triggered PP2A degradation through the ubiquitin–proteasome system, leading to intraneuronal accumulation of p‐Tau. Pharmacologically promoting cholesterol efflux alleviated tau‐related neuropathologies and improved cognition in APOE4‐HFD mice. These findings established a causal link through APOE4 between AS and tauopathies.

Emerging evidence suggests that AS may contribute to the development and progression of AD. The compromised cerebral blood flow resulting from atherosclerotic changes could exacerbate neuronal damage and trigger neuroinflammation [27], both of which might promote the development of AD pathologies like Aβ deposition and p‐Tau accumulation [28, 29, 30]. A recent study showed that asparagine endopeptidase signaling coupled AS and AD pathology, and inactivation of this signaling pathway protected from the AS and AD processes [31]. Furthermore, many shared risk factors such as hypertension, diabetes, and dyslipidemia were all known to influence the pathogenesis of both AS and AD [32, 33], suggesting a common underlying mechanism. Besides, proteomic and single‐cell RNA sequencing also unraveled that many AD‐related molecular changes occurred in brain tissues of AS patients [34]. It has been widely revealed that APOE4 is a common risk factor for both AS and AD; it induces variable pathological changes compared with the wild‐type APOE3 and protective APOE2 variant [35]. Indeed, we found here that APOE4 carriers had a higher AS plaque, more severe p‐Tau accumulation, glial activation, and neurodegeneration in AS patients and mice. Consistent with these results, a recent study also showed that more severe neuritic tau pathology occurred in Aβ‐affected brain regions of sporadic AD patients with the APOE4 genotype by analyzing the p‐Tau interactome [36]. Significant increases in both cerebrospinal fluid and plasma p‐Tau levels were also reported in *APOE4/4* AD patients [37]. It should be noted that APOE4 can promote tau hyperphosphorylation and accumulation, as well as facilitate neuroinflammation independent of Aβ [12], which might help explain why we did not observe Aβ changes in the present study.

Many studies have showed that cerebral cholesterol plays critical roles in the regulation of neurological functions [5, 22, 38]. In the primary cultured neurons experimental test, we did not find any difference in PP2A B and PP2A C mRNAs among APOE3 and APOE4 neurons treated with cholesterol. We thought that the reduction of PP2A induced by cholesterol was in a posttranslational dependent manner. We here investigated in AS brains the effect of APOE4 on PP2A, which plays critical roles in the modulation of tau dephosphorylation [39], and found that both APOE4^+^ AS patients and APOE3‐HFD mice exhibited lower PP2A, mechanistically due to the facilitation of ubiquitin–proteasome system‐mediated proteolysis of PP2A by aberrant accumulation of cholesterol. Moreover, elevated cholesterol levels may also exacerbate tau pathology through enhancing the aggregation and toxicity of Aβ [40], which was capable of mediating disruption of calcium homeostasis and subsequently activating phospholipases to lead to tau hyperphosphorylation [41]. This was supported by the finding that cholesterol‐rich lipid rafts are implicated in the co‐localization of tau with Aβ [42]. Besides, activation of kinases involved in tau phosphorylation, such as CDK5 and GSK3β, on cellular membranes may also be influenced by cholesterol levels [43]. However, there were also studies suggesting that the reduction of intraneuronal cholesterol promoted the seeding and propagation of tau [44]. In our study, we employed HP‐β‐CD, which could promote cholesterol efflux, to illustrate whether aiding cholesterol could exert a protective effect in APOE4 AS mice. After subcutaneous injections of HP‐β‐CD for 8 weeks, the APOE4 mice showed a lower cerebral cholesterol content compared with the sham group. Moreover, the AS pathology, glial activation, tau pathology, synaptic degeneration, and cognitive impairment were alleviated by HP‐β‐CD. Therefore, the exact role of cholesterol homeostasis in determining the hyperphosphorylation/accumulation of tau deserves further investigation.

In conclusion, our study indicated that cholesterol couples AS and AD‐like tau pathology in APOE4 carriers. Promoting cholesterol homeostasis was effective in alleviating tau pathology and decreasing the risk for AD in AS patients of APOE4 genotype. These findings informed new therapeutic targets for AS and AD.

## Author Contributions

Xiaolan Qi, Bing Xia, and Jie Zheng conceived the research project and provided supervision; Jiuyang Ding, Baofei Sun, and Yang Gao established assays and performed experiments; Jiuyang Ding and Cihang Gu analyzed data; Xiaolan Qi and Jian Zhang provided technical and scientific support; Jiuyang Ding and Li Wang drafted the manuscript; Jiuyang Ding, Bing Xia, and Baofei Sun provided resources; all authors agreed on the final version of the paper.

## Consent

Written informed consent was obtained from family members of all deceasedindividuals.

## Conflicts of Interest

The authors declare no conflicts of interest.

## Supporting information


Data S1

## Data Availability

The data that support the findings of this study are available on request from the corresponding author. The data are not publicly available due to privacy or ethical restrictions.
