# Supplementary material for: APOE4 Exacerbates Cerebral Tau Pathology Through Cholesterol‐Induced Degradation of Phosphatase in Atherosclerosis
Source: CNS Neurosci Ther. 2025 Jul 30;31(8):e70536. doi: 10.1111/cns.70536 (PMC12311220; doi:10.1111/cns.70536)

Supplementary materials

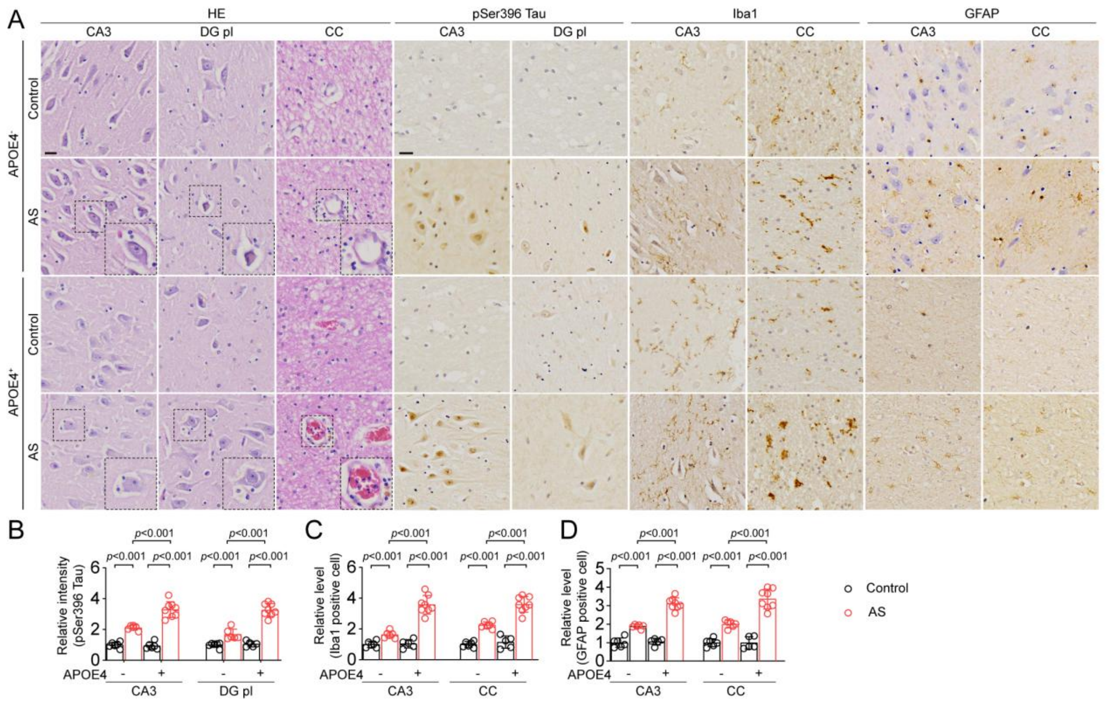

Figure S1. APOE4 promotes tau hyperphosphorylation and glial activation in several other brain regions of AS patients.

APOE4$^+$ AS patients had prominent neuronophagia (A, left), more potent increase of pSer396 tau (B), iba1-labeled active microglia (C), and GFAP-labeled active astrocytes (D) in the CA3 and DG polymorph layer (pl), and blood vessel cuffing in corpus collosum (CC). Bar, 20 µm. Two-way ANOVA followed by Bonferroni's *post hoc* tests. n = 5~8 in each group.

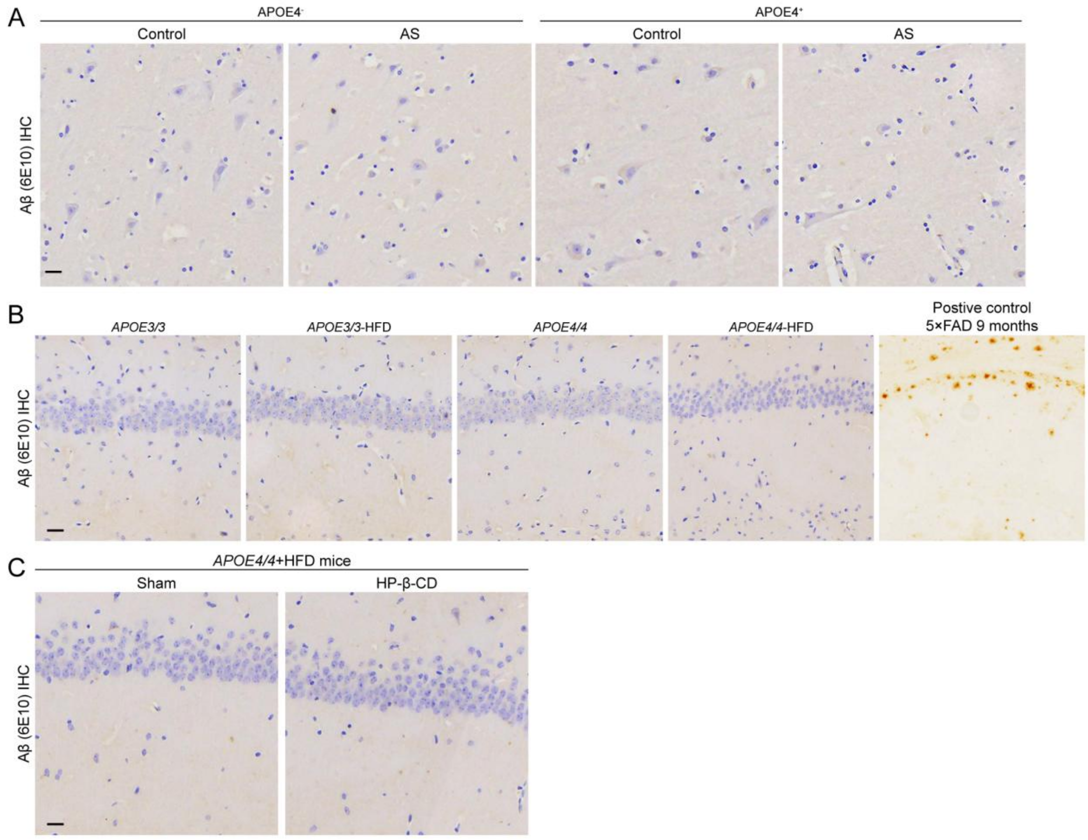

Figure S2. No Aβ plaque was detected in postmortem AS brains and AS mice brains.

No Aβ plaque was detected by 6E10 IHC staining of postmortem AS hippocampal tissues (A), HFD-treated APOE3 or APOE4 mice (B), and HP-β-CD-treated APOE4-HFD mice (C). A brain slice of 9-month old 5×FAD mice was stained as a positive control. Bar, 20 μm.

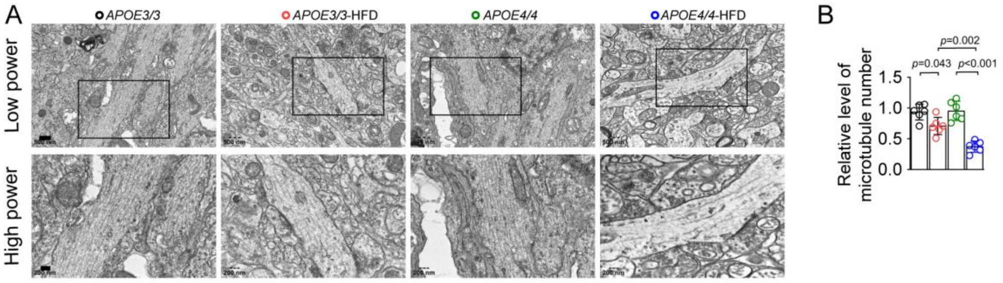

Figure S3. APOE4 exacerbates microtubule depolymerization in HFD-treated mice.

(A) Representative TEM images of microtubule in hippocampal CA1 neurons.

(B) Statistical results of microtubule bundles. Two-way ANOVA followed by Bonferroni's *post hoc* tests. n = 6 mice per group. Bar, 500 nm in upper panels, 200 nm in lower panels.

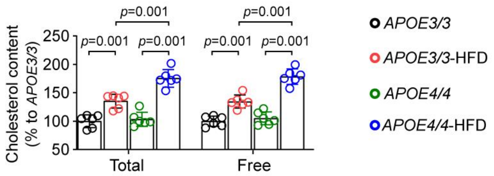

Figure S4. More potent increase of total and free cholesterol content in the serum of HFD-treated APOE4 mice. Cholesterol levels in serum were measured by ELISA. Two-way ANOVA followed by Bonferroni's *post hoc* tests. n = 6 mice per group.

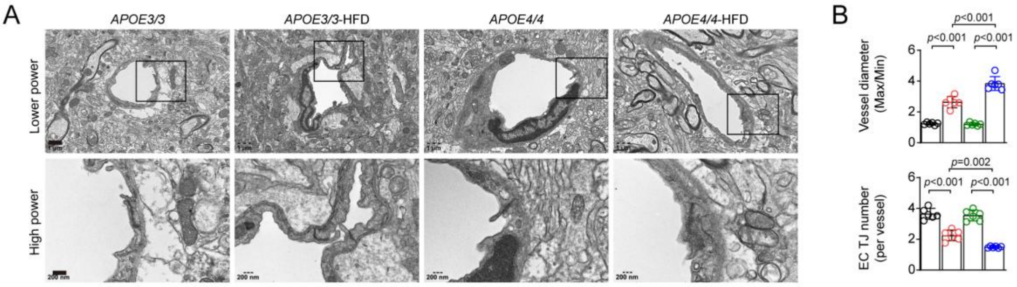

Figure S5. APOE4 promoted BBB breakdown in AS mice.

(A) Representative TEM images of BBB in CA1 region of mice.

(B) Quantification of vessels diameter and number of tight junctions in BBB capillary. Two-way ANOVA followed by Bonferroni' *post hoc* tests. n = 6 mice per group. Bar, 500 nm in upper panels, 200 nm in lower panels.

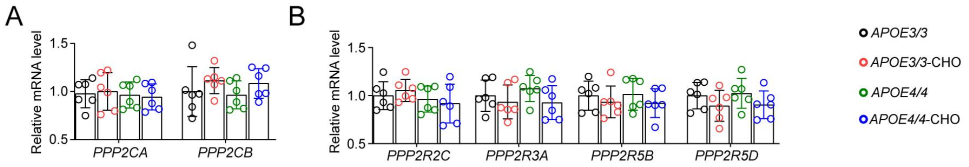

Figure S6. APOE4 and cholesterol treatment did not change mRNA levels of PP2A subunits.

(A) APOE4 and cholesterol treatment did not change mRNA levels of *PPP2CA* and *PPP2CB*. Two-way ANOVA followed by Bonferroni's *post hoc* tests. n = 6 mice per group.

(B) APOE4 and cholesterol treatment did not change mRNA levels of *PPP2R2C*, *PPP2R3A*, *PPP2R5B and PPP2R5D*. Two-way ANOVA followed by Bonferroni's *post hoc* tests. n = 6 mice per group.

Table S1. Information of human samples

Group	Gender	Age (year)	Comorbidity	COD	PMI (hrs)
APOE4^{-} control	F	46	NA	Car accident	49
APOE4^{-} control	M	49	NA	Hemorrhagic shock	52
APOE4^{-} control	M	54	NA	Hemorrhagic shock	38
APOE4^{-} control	F	71	NA	Fall	40
APOE4^{-} control	F	69	NA	Pulmonary embolism	18
APOE4^{-} control	M	78	Hypertension	MODS	24
APOE4^{-} AS	M	63	Coronary atherosclerosis	Acute myocardial infarction	32
APOE4^{-} AS	F	56	Coronary atherosclerosis	Acute myocardial infarction	24
APOE4^{-} AS	F	73	Coronary atherosclerosis	Acute myocardial infarction	19
APOE4^{-} AS	F	42	Coronary atherosclerosis, COVID-19 inection	MODS	18
APOE4^{-} AS	M	56	Coronary atherosclerosis	MODS	22
APOE4^{-} AS	M	69	Coronary atherosclerosis	Acute myocardial infarction	29
APOE4^{+} control	M	63	NA	Fall	32
APOE4^{+} control	F	84	COVID19, hypertension	MODS	37
APOE4^{+} control	M	63	Covid19	Shock	42
APOE4^{+} control	F	69	COVID19, Diabetes mellitus	Pulmonary embolism	43
APOE4^{+} control	F	73	Hypertension	Heart failure	29
APOE4^{+} AS	M	63	Coronary atherosclerosis,Hypertension,	Acute myocardial infarction	39
APOE4^{+} AS	M	68	Coronary atherosclerosis, Nephritis, COVID-19 inection	Heart failure	44
APOE4^{+} AS	M	54	Coronary atherosclerosis	Multiple organ dysfunction syndrome	39
APOE4^{+} AS	F	49	Coronary atherosclerosisHypertension,	Acute myocardial infarction	51
APOE4^{+} AS	M	79	Coronary atherosclerosis, Hypertension	Acute myocardial infarction	29
APOE4^{+} AS	F	83	Coronary atherosclerosis,Hypertension	Heart failure	37
APOE4^{+} AS	F	87	Coronary atherosclerosis,Hypertension	Acute myocardial infarction	38
APOE4^{+} AS	F	62	Coronary atherosclerosis,Hypertension	Acute myocardial infarction	43

Abbreviations: Gender: F, female; M, male. NA, non-applicable. COD, cause of death. PMI, postmortem interval.

Table S2. Antibodies used in this study

Antibodies	Source	Identifier	Dilution

AT8	ThermoFisher Scientific	Cat#MN1020	WB 1:1000; IHC, 1:200
Phospho-Tau Ser396	ThermoFisher Scientific	Cat#44-752G	WB, 1:1000; IHC, 1:200
Phospho-Tau Thr217	ThermoFisher Scientific	Cat#44-744	WB, 1:1000; IHC, 1:200
Phospho-Tau Thr231	ThermoFisher Scientific	Cat#44-746G	WB, 1:1000; IHC, 1:200
Phospho-Tau Thr181	Cell Signaling Technology	Cat#12885	WB, 1:1000; IHC, 1:200
Phospho-Tau Ser404	Cell Signaling Technology	Cat#20194	WB, 1:1000
Tau5	Abcam	Cat#20194	WB, 1:1000
VAMP2	Cell Signaling Technology	Cat#13508	WB, 1:1000
PSD95	Cell Signaling Technology	Cat#2507	WB, 1:1000
Synaptophysin	Cell Signaling Technology	Cat#36406	WB, 1:1000
NMDA Receptor 2A	Cell Signaling Technology	Cat#4205	WB, 1:1000
NMDA Receptor 2B	Cell Signaling Technology	Cat#4212	WB, 1:1000
PP2A B	Cell Signaling Technology	Cat#4953	WB, 1:1000; Co-IP, 1:200
PP2A C	Cell Signaling Technology	Cat#2038	WB, 1:1000; Co-IP, 1:200
Ubiquitin	Cell Signaling Technology	Cat#20326	WB, 1:1000
GAPDH	Cell Signaling Technology	Cat#2118	WB, 1:1000
GFAP	Cell Signaling Technology	Cat#3670	IHC, 1:200
Iba1	Cell Signaling Technology	Cat#ab178846	IHC, 1:300
NeuN	Abcam	Cat#ab104224	IHC, 1:200
MAP2	Abcam	Cat#ab254264	IF, 1:200
donkey anti-rabbit Alexa Fluor 555	ThermoFisher Scientific	Cat#A-31572	IF, 1:500

Table S3. Summary of the statistical analyses used in this study

Figure	Sample size	Statistical methods	F/T/r and p value
1B	$APOE4^-$ Control = 6<br>$APOE4^-$ AS = 6<br>$APOE4^+$ Control = 5<br>$APOE4^+$ AS = 8	Two-way ANOVA	Phenotype, F (1, 21) = 154.39, p < 0.001,<br>Genotype, F (1, 21) = 12.659, p = 0.002,<br>Phenotype × Genotype, F (1, 21) = 12.659, p = 0.002.
1C	$APOE4^-$ Control = 6<br>$APOE4^-$ AS = 6<br>$APOE4^+$ Control = 5<br>$APOE4^+$ AS = 8	Two-way ANOVA	CA1: Phenotype, F (1, 21) = 70.029, p < 0.001, Genotype, F (1, 21) = 15.894, p = 0.001, Phenotype × Genotype, F (1, 21) = 13.384, p = 0.001.<br>DG gl: Phenotype, F (1, 21) = 121.437, p < 0.001, Genotype, F (1, 21) = 56.274, p < 0.001, Phenotype × Genotype, F (1, 21) = 48.446, p < 0.001.<br>EC: Phenotype, F (1, 21) = 231.5, p < 0.001, Genotype, F (1, 21) = 54.3034, p < 0.0011, Phenotype × Genotype, F (1, 21) = 49.897, p < 0.001.
1D	$APOE4^-$ Control = 6<br>$APOE4^-$ AS = 6<br>$APOE4^+$ Control = 5<br>$APOE4^+$ AS = 8	Two-way ANOVA	CA1: Phenotype, F (1, 21) = 243.993, p < 0.001, Genotype, F (1, 21) = 39.534, p < 0.001, Phenotype × Genotype, F (1, 21) = 34.384, p < 0.001.<br>DG gl: Phenotype, F (1, 21) = 133.708, p < 0.001, Genotype, F (1, 21) = 41.567, p < 0.001, Phenotype × Genotype, F (1, 21) = 31.929, p < 0.001.<br>EC: Phenotype, F (1, 21) = 95.329, p < 0.001, Genotype, F (1, 21) = 21.913, p < 0.0011, Phenotype × Genotype, F (1, 21) = 16.331, p <

			0.001.
1E	APOE4⁻ Control = 6<br>APOE4⁻ AS = 6<br>APOE4⁺ Control = 5<br>APOE4⁺ AS = 8	Two-way ANOVA	CA1: Phenotype, F (1, 21) = 161.807, p < 0.001, Genotype, F (1, 21) = 57.233, p < 0.001, Phenotype × Genotype, F (1, 21) = 43.588, p < 0.001.<br>DG gl: Phenotype, F (1, 21) = 223.445, p < 0.001, Genotype, F (1, 21) = 26.668, p < 0.001, Phenotype × Genotype, F (1, 21) = 19.612, p < 0.001.<br>EC: Phenotype, F (1, 21) = 147.874, p < 0.001, Genotype, F (1, 21) = 43.096, p < 0.001, Phenotype × Genotype, F (1, 21) = 24.474, p < 0.001.
1G	APOE4⁻ Control = 6<br>APOE4⁻ AS = 6<br>APOE4⁺ Control = 5<br>APOE4⁺ AS = 8	Two-way ANOVA	Phenotype, F (1, 21) = 80.327, p < 0.001, Genotype, F (1, 21) = 43.096, p < 0.001, Phenotype × Genotype, F (1, 21) = 24.474, p < 0.001
1H	APOE4⁻ Control = 6<br>APOE4⁻ AS = 6<br>APOE4⁺ Control = 5<br>APOE4⁺ AS = 8	Two-way ANOVA	Phenotype, F (1, 21) = 81.943, p < 0.001, Genotype, F (1, 21) = 9.598, p = 0.005, Phenotype × Genotype, F (1, 21) = 11.221, p = 0.003
1I	APOE4⁻ Control = 6<br>APOE4⁻ AS = 6<br>APOE4⁺ Control = 5<br>APOE4⁺ AS = 8	Two-way ANOVA	Phenotype, F (1, 21) = 70.367, p < 0.001, Genotype, F (1, 21) = 15.455, p = 0.001, Phenotype × Genotype, F (1, 21) = 14.61, p = 0.001
1J	APOE4⁻ Control = 6<br>APOE4⁻ AS = 6<br>APOE4⁺ Control = 5<br>APOE4⁺ AS = 8	Two-way ANOVA	Phenotype, F (1, 21) = 0.849, p = 0.367, Genotype, F (1, 21) = 0.048, p = 0.829, Phenotype × Genotype, F (1, 21) = 0.364, p = 0.552
1K	APOE4⁻ AS=6<br>APOE4⁺ AS = 8	Pearson correlation analysis	r = 0.8162, p = 0.0004
1L	APOE4⁻ AS = 6<br>APOE4⁺ AS = 8	Pearson correlation analysis	r = 0.8372, p = 0.0002
1M	APOE4⁻ AS = 6<br>APOE4⁺ AS = 8	Pearson correlation analysis	r = 0.7436, p = 0.0023
1O	APOE4⁻ Control = 6<br>APOE4⁻ AS = 6<br>APOE4⁺ Control = 5<br>APOE4⁺ AS = 8	Two-way ANOVA	Phenotype, F (1, 21) = 134.263, p < 0.001, Genotype, F (1, 21) = 0.674, p = 0.421, Phenotype × Genotype, F (1, 21) = 4.152, p = 0.054
2B	APOE3/3 = 6<br>APOE3/3+HFD = 6<br>APOE4/4 = 6<br>APOE4/4+HFD = 6	Two-way ANOVA	pSer396 Tau, Genotype, F (1, 20) = 103.749, p < 0.001, Treatment, F (1, 20) = 27.102, p < 0.001, Genotype × Treatment, F (1, 20) = 21.743, p < 0.001<br>pThr231 Tau, Genotype, F (1, 20) = 224.736, p < 0.001, Treatment, F (1, 20) = 55.579, p < 0.001, Genotype × Treatment, F (1, 20) = 48.894, p < 0.001<br>pThr181 Tau, Genotype, F (1, 20) = 107.549, p < 0.001, Treatment, F (1, 20) = 21.284, p < 0.001, Genotype × Treatment, F (1, 20) = 13.782, p < 0.001

			pSer404 Tau, Genotype, $F (1, 20) = 706.984$, $p < 0.001$, Treatment, $F (1, 20) = 58.893$, $p < 0.001$, Genotype × Treatment, $F (1, 20) = 57.309$, $p < 0.001$<br>AT8, Genotype, $F (1, 20) = 185.816$, $p < 0.001$, Treatment, $F (1, 20) = 21.284$, $p < 0.001$, Genotype × Treatment, $F (1, 20) = 13.782$, $p < 0.001$<br>pThr217 Tau, Genotype, $F (1, 20) = 30.42$, $p < 0.001$, Treatment, $F (1, 20) = 14.949$, $p = 0.001$, Genotype × Treatment, $F (1, 20) = 13.782$, $p < 0.001$<br>total Tau, Genotype, $F (1, 20) = 0.064$, $p = 0.802$, Treatment, $F (1, 20) = 0.157$, $p = 0.696$, Genotype × Treatment, $F (1, 20) = 0.789$, $p = 0.385$
2D	APOE3/3 = 6<br>APOE3/3+HFD = 6<br>APOE4/4 = 6<br>APOE4/4+HFD = 6	Two-way ANOVA	Genotype, $F (1, 20) = 687.978$, $p < 0.001$, Treatment, $F (1, 20) = 112.685$, $p < 0.001$, Genotype × Treatment, $F (1, 20) = 112.685$, $p < 0.001$
2E	APOE3/3 = 6<br>APOE3/3+HFD = 6<br>APOE4/4 = 6<br>APOE4/4+HFD = 6	Two-way ANOVA	Genotype, $F (1, 20) = 447.578$, $p < 0.001$, Treatment, $F (1, 20) = 36.523$, $p < 0.001$, Genotype × Treatment, $F (1, 20) = 27.366$, $p < 0.001$
2F	APOE3/3 = 6<br>APOE3/3+HFD = 6<br>APOE4/4 = 6<br>APOE4/4+HFD = 6	Two-way ANOVA	Genotype, $F (1, 20) = 254.378$, $p < 0.001$, Treatment, $F (1, 20) = 42.903$, $p < 0.001$, Genotype × Treatment, $F (1, 20) = 33.823$, $p < 0.001$
2G	APOE3/3 = 6<br>APOE3/3+HFD = 6<br>APOE4/4 = 6<br>APOE4/4+HFD = 6	Two-way ANOVA	Genotype, $F (1, 20) = 2.018$, $p = 0.171$, Treatment, $F (1, 20) = 0.315$, $p = 0.581$, Genotype × Treatment, $F (1, 20) = 1.337$, $p = 0.261$
2H	APOE3/3 = 6<br>APOE3/3+HFD = 6<br>APOE4/4 = 6<br>APOE4/4+HFD = 6	Two-way ANOVA	PThr 231 Tau, Genotype, $F (1, 20) = 801.811$, $p < 0.001$, Treatment, $F (1, 20) = 231.211$, $p < 0.001$, Genotype × Treatment, $F (1, 20) = 194.439$, $p < 0.001$<br>pSer396 Tau, Genotype, $F (1, 20) = 463.547$, $p < 0.001$, Treatment, $F (1, 20) = 113.693$, $p < 0.001$, Genotype × Treatment, $F (1, 20) = 103.453$, $p < 0.001$<br>pThr217 Tau, Genotype, $F (1, 20) = 262.838$, $p < 0.001$, Treatment, $F (1, 20) = 51.919$, $p < 0.001$, Genotype × Treatment, $F (1, 20) = 35.93$, $p < 0.001$<br>pThr181 Tau, Genotype, $F (1, 20) = 1129.744$, $p < 0.001$, Treatment, $F (1, 20) = 190.54$, $p < 0.001$, Genotype × Treatment, $F (1, 20) = 147.963$, $p < 0.001$<br>AT8: Genotype, $F (1, 20) = 431.824$, $p < 0.001$, Treatment, $F (1, 20) = 84.569$, $p < 0.001$, Genotype × Treatment, $F (1, 20) = 54.439$, $p < 0.001$
3B	APOE3/3 = 6<br>APOE3/3+HFD = 6<br>APOE4/4 = 6	Two-way ANOVA	VAMP2, Genotype, $F (1, 20) = 102.882$, $p < 0.001$, Treatment, $F (1, 20) = 8.814$, $p = 0.008$, Genotype × Treatment, $F (1, 20) = 23.022$, $p < 0.001$

	APOE4/4+HFD = 6		PSD95, Genotype, F (1, 20) = 87.53, p < 0.001, Treatment, F (1, 20) = 9.398, p = 0.006, Genotype × Treatment, F (1, 20) = 29.042, p < 0.001<br>NR2A, Genotype, F (1, 20) = 120.447, p < 0.001, Treatment, F (1, 20) = 21.615, p < 0.001, Genotype × Treatment, F (1, 20) = 46.179, p < 0.001<br>NR2B, Genotype, F (1, 20) = 171.368, p < 0.001, Treatment, F (1, 20) = 33.447, p < 0.001, Genotype × Treatment, F (1, 20) = 38.322, p < 0.001
3D	APOE3/3 = 6<br>APOE3/3+HFD = 6<br>APOE4/4 = 6<br>APOE4/4+HFD = 6	Two-way ANOVA	80 μm, Genotype, F = 85.842, p < 0.001, Treatment, F = 9.993, p = 0.005, Genotype × Treatment, F = 5.094, p = 0.035<br>120 μm, Genotype, F = 95.021, p < 0.001, Treatment, F = 21.323, p < 0.001, Genotype × Treatment, F = 3.971, p = 0.06
3E	APOE3/3 = 6<br>APOE3/3+HFD = 6<br>APOE4/4 = 6<br>APOE4/4+HFD = 6	Two-way ANOVA	Genotype, F (1, 20) = 52.691, p < 0.001, Treatment, F (1, 20) = 5.269, p = 0.033, Genotype × Treatment, F (1, 20) = 2.889, p = 0.105
3F	APOE3/3 = 6<br>APOE3/3+HFD = 6<br>APOE4/4 = 6<br>APOE4/4+HFD = 6	Two-way ANOVA	Genotype, F (1, 20) = 115.238, p < 0.001, Treatment, F (1, 20) = 10.917, p = 0.004, Genotype × Treatment, F (1, 20) = 0.849, p = 0.368
3G	APOE3/3 = 12<br>APOE3/3+HFD = 12<br>APOE4/4 = 12<br>APOE4/4+HFD = 12	Two-way ANOVA	Genotype, F (1, 20) = 24.59, p < 0.001, Treatment, F (1, 20) = 113.96, p < 0.001, Genotype × Treatment, F (1, 20) = 11.859, p < 0.001
3H	APOE3/3 = 12<br>APOE3/3+HFD = 12<br>APOE4/4 = 12<br>APOE4/4+HFD = 12	Two-way ANOVA	Genotype, F (1, 20) = 40.735, p < 0.001, Treatment, F (1, 20) = 257.174, p < 0.001, Genotype × Treatment, F (1, 20) = 14.3, p < 0.001
3I	APOE3/3 = 12<br>APOE3/3+HFD = 12<br>APOE4/4 = 12<br>APOE4/4+HFD = 12	Two-way ANOVA	Genotype, F (1, 20) = 45.973, p < 0.001, Treatment, F (1, 20) = 254.301, p < 0.001, Genotype × Treatment, F (1, 20) = 17.611, p < 0.001
4B	APOE3/3 = 6<br>APOE3/3+HFD = 6<br>APOE4/4 = 6<br>APOE4/4+HFD = 6	Two-way ANOVA	Genotype, F (1, 20) = 94.855, p < 0.001, Treatment, F (1, 20) = 15.738, p = 0.004, Genotype × Treatment, F (1, 20) = 3.175, p = 0.09
4D	APOE3/3 = 6<br>APOE3/3+CHO = 6<br>APOE4/4 = 6<br>APOE4/4+CHO = 6	Two-way ANOVA	pSer396 Tau, Genotype, F (1, 20) = 62.135, p < 0.001, Treatment, F (1, 20) = 20.955, p < 0.001, Genotype × Treatment, F (1, 20) = 2.137, p = 0.159<br>AT8, Genotype, F (1, 20) = 46.899, p < 0.001, Treatment, F (1, 20) = 19.633, p < 0.001, Genotype × Treatment, F (1, 20) = 11.391, p = 0.003<br>total Tau, Genotype, F (1, 20) = 0.202, p = 0.658, Treatment, F (1, 20) = 1.98, p = 0.175, Genotype × Treatment, F (1, 20) = 2.354, p = 0.141

4E	APOE3/3 = 6<br>APOE3/3+CHO = 6<br>APOE4/4 = 6<br>APOE4/4+CHO = 6	Two-way ANOVA	PSD95, Genotype, $F(1, 20) = 384.912$, $p < 0.001$, Treatment, $F(1, 20) = 63.115$, $p < 0.001$, Genotype × Treatment, $F(1, 20) = 26.011$, $p < 0.001$<br>NR2A, Genotype, $F(1, 20) = 88.51$, $p < 0.001$, Treatment, $F(1, 20) = 13.194$, $p = 0.002$, Genotype × Treatment, $F(1, 20) = 5.167$, $p = 0.034$<br>NR2B, Genotype, $F(1, 20) = 57.678$, $p < 0.001$, Treatment, $F(1, 20) = 8.276$, $p = 0.009$, Genotype × Treatment, $F(1, 20) = 2.382$, $p = 0.138$
4G	APOE3/3 = 6<br>APOE3/3+CHO = 6<br>APOE4/4 = 6<br>APOE4/4+CHO = 6	Two-way ANOVA	80 μm, Genotype, $F = 131.507$, $p < 0.001$, Treatment, $F = 15.306$, $p = 0.001$, Genotype × Treatment, $F = 11.469$, $p = 0.003$<br>120 μm, Genotype, $F = 124.612$, $p < 0.001$, Treatment, $F = 11.991$, $p = 0.002$, Genotype × Treatment, $F = 4.959$, $p = 0.038$
4H	APOE3/3 = 6<br>APOE3/3+CHO = 6<br>APOE4/4 = 6<br>APOE4/4+CHO = 6	Two-way ANOVA	Genotype, $F = 164.956$, $p < 0.001$, Treatment, $F = 23.013$, $p < 0.001$, Genotype × Treatment, $F = 4.338$, $p = 0.05$
4I	APOE3/3 = 6<br>APOE3/3+CHO = 6<br>APOE4/4 = 6<br>APOE4/4+CHO = 6	Two-way ANOVA	Genotype, $F = 132.805$, $p < 0.001$, Treatment, $F = 24.497$, $p < 0.001$, Genotype × Treatment, $F = 12.577$, $p = 0.002$
5B	APOE3/3 = 6<br>APOE3/3+CHO = 6<br>APOE4/4 = 6<br>APOE4/4+CHO = 6	Two-way ANOVA	PP2A B, Genotype, $F(1, 20) = 89.457$, $p < 0.001$, Treatment, $F(1, 20) = 1.662$, $p = 0.212$, Genotype × Treatment, $F(1, 20) = 14.218$, $p = 0.001$<br>PP2A C, Genotype, $F(1, 20) = 209.792$, $p < 0.001$, Treatment, $F(1, 20) = 28.217$, $p < 0.001$, Genotype × Treatment, $F(1, 20) = 31.934$, $p < 0.001$
5E	APOE3/3 = 6<br>APOE3/3+CHO = 6<br>APOE4/4 = 6<br>APOE4/4+CHO = 6	Two-way ANOVA	Ub/PP2A B, Genotype, $F(1, 20) = 186.892$, $p < 0.001$, Treatment, $F(1, 20) = 18.733$, $p < 0.001$, Genotype × Treatment, $F(1, 20) = 11.655$, $p = 0.003$<br>Ub/PP2A C, Genotype, $F(1, 20) = 343.161$, $p < 0.001$, Treatment, $F(1, 20) = 80.564$, $p < 0.001$, Genotype × Treatment, $F(1, 20) = 57.863$, $p < 0.001$
5G	Sham = 6<br>MG132 = 6	Independent-samples T test	PP2A B, $T = 39.966$, $p < 0.001$<br>PP2A C, $T = 9.941$, $p = 0.01$
5H	Sham = 6<br>MG132 = 6	Independent-samples T test	pSer396 Tau, $T = 52.584$, $p < 0.001$<br>AT8, $T = 115.54$, $p < 0.001$<br>pThr217 Tau, $T = 27.806$, $p < 0.001$<br>Total Tau, $T = 0.799$, $p = 0.392$
5J	$APOE4^-$ Control=6<br>$APOE4^-$ AS=6<br>$APOE4^+$ Control=5<br>$APOE4^+$ AS=8	Two-way ANOVA	PP2A B, Phenotype, $F(1, 21) = 61.046$, $p < 0.001$, Genotype, $F(1, 21) = 36.583$, $p < 0.001$, Phenotype × Genotype, $F(1, 21) = 7.001$, $p = 0.015$,
5K	$APOE4^-$ Control = 6<br>$APOE4^-$ AS = 6<br>$APOE4^+$ Control = 5	Two-way ANOVA	PP2A C, Phenotype, $F(1, 21) = 103.174$, $p < 0.001$, Genotype, $F(1, 21) = 13.653$, $p = 0.001$, Phenotype × Genotype, $F(1, 21) = 6.27$, $p = 0.021$

	APOE4⁺ AS = 8		
5M	APOE3/3 = 6<br>APOE3/3+HFD = 6<br>APOE4/4 = 6<br>APOE4/4+HFD = 6	Two-way ANOVA	PP2A B, Phenotype, $F (1, 20) = 61.627, p < 0.001$, Genotype, $F (1, 20) = 13.485, p = 0.002$, Phenotype × Genotype, $F (1, 20) = 8.289, p = 0.009$<br>PP2A C, Phenotype, $F (1, 20) = 110.403, p < 0.001$, Genotype, $F (1, 20) = 10.779, p = 0.004$, Phenotype × Genotype, $F (1, 20) = 13.965, p = 0.001$
6B	Sham = 6<br>HP-β-CD = 6	Independent-samples T test	$T = 3.364, p = 0.007$
6C	Sham = 6<br>HP-β-CD = 6	Independent-samples T test	$T = 3.273, p = 0.008$
6D	Sham = 6<br>HP-β-CD = 6	Independent-samples T test	$T = 2.413, p = 0.036$
6F	Sham = 6<br>HP-β-CD = 6	Independent-samples T test	$T = 23.659, p = 0.001$
6G	Sham = 6<br>HP-β-CD = 6	Independent-samples T test	pThr231 Tau, $T = 23.659, p = 0.001$<br>pSer396 Tau, $T = 52.956, p < 0.001$<br>pThr217 Tau, $T = 169.403, p < 0.001$<br>pThr181 Tau, $T = 22.474, p < 0.001$<br>AT8, $T = 12.097, p = 0.006$
6H	Sham = 6<br>HP-β-CD = 6	Independent-samples T test	GFAP, $T = 65.553, p < 0.001$<br>Iba1, $T = 176.005, p < 0.001$<br>NeuN, $T = 0.177, p = 0.683$
6J	Sham = 6<br>HP-β-CD = 6	Independent-samples T test	pSer396 Tau, $T = 71.254, p < 0.001$<br>AT8, $T = 82.183, p < 0.001$<br>pThr231 Tau, $T = 67.621, p < 0.001$<br>pThr181 Tau, $T = 9.523, p =0.012$<br>pThr217 Tau, $T = 122.113, p < 0.001$<br>pSer404 Tau, $T = 17.078, p= 0.002$<br>Total Tau, $T = 1.336, p = 0.275$
6L	Sham = 6<br>HP-β-CD = 6	Independent-samples T test	PP2A B, $T = 28.339, p < 0.001$<br>PP2A C, $T = 6.075, p = 0.033$<br>Ub, $T = 88.247, p < 0.001$<br>Syn, $T = 49.027, p < 0.001$
6N	Sham = 6<br>HP-β-CD = 6	Independent-samples T test	40 μm, $T = 4.812, p < 0.001$<br>80 μm, $T = 6.434, p < 0.001$<br>120 μm, $T = 7.265, p < 0.001$<br>160 μm, $T = 6.742, p < 0.001$<br>200 μm, $T = 3.229, p = 0.009$
6O	Sham = 6<br>HP-β-CD = 6	Independent-samples T test	$T = 29.518, p < 0.001$
6P	Sham = 6<br>HP-β-CD = 6	Independent-samples T test	$T = 263.322, p < 0.001$
6Q	Sham = 12<br>HP-β-CD = 12	Independent-samples T test	$T = 3.73, p < 0.004$
6R	Sham = 12	Independent-samples T	Escape latency,

	HP-β-CD = 12	test	Day 3, T = 3.849, $p = 0.003$<br>Day4, T = 5.813, $p < 0.001$<br>Day5, T = 5.369, $p < 0.001$<br>Time in quadrant, T = 5.155, $p < 0.001$
S1B	$APOE4^-$ Control = 6<br>$APOE4^-$ AS = 6<br>$APOE4^+$ Control = 5<br>$APOE4^+$ AS = 8	Two-way ANOVA	CA3, Phenotype, $F (1, 21) = 181.766$, $p < 0.001$, Genotype, $F (1, 21) = 18.884$, $p < 0.001$, Phenotype × Genotype, $F (1, 21) = 23.387$, $p < 0.001$<br>DG pl, Phenotype, $F (1, 21) = 122.641$, $p < 0.001$, Genotype, $F (1, 21) = 41.28$, $p < 0.001$, Phenotype × Genotype, $F (1, 21) = 35.305$, $p < 0.001$
S1C	$APOE4^-$ Control = 6<br>$APOE4^-$ AS = 6<br>$APOE4^+$ Control = 5<br>$APOE4^+$ AS = 8	Two-way ANOVA	CA3, Phenotype, $F (1, 21) = 97.938$, $p < 0.001$, Genotype, $F (1, 21) = 38.342$, $p < 0.001$, Phenotype × Genotype, $F (1, 21) = 35.312$, $p < 0.001$<br>CC, Phenotype, $F (1, 21) = 152.771$, $p < 0.001$, Genotype, $F (1, 21) = 24.512$, $p < 0.001$, Phenotype × Genotype, $F (1, 21) = 15.816$, $p < 0.001$
S1D	$APOE4^-$ Control = 6<br>$APOE4^-$ AS = 6<br>$APOE4^+$ Control = 5<br>$APOE4^+$ AS = 8	Two-way ANOVA	CA3, Phenotype, $F (1, 21) = 228.602$, $p < 0.001$, Genotype, $F (1, 21) = 43.284$, $p < 0.001$, Phenotype × Genotype, $F (1, 21) = 36.779$, $p < 0.001$<br>CC, Phenotype, $F (1, 21) = 127.314$, $p < 0.001$, Genotype, $F (1, 21) = 20.42$, $p < 0.001$, Phenotype × Genotype, $F (1, 21) = 20.906$, $p < 0.001$
S2B	APOE3/3 = 6<br>APOE3/3+HFD = 6<br>APOE4/4 = 6<br>APOE4/4+HFD = 6	Two-way ANOVA	genotype, $F (1, 21) = 8.647$, $p = 0.008$, treatment, $F (1, 21) = 56.577$, $p < 0.001$, phenotype × genotype, $F (1, 21) = 10.766$, $p = 0.004$
S3	APOE3/3 = 6<br>APOE3/3+HFD = 6<br>APOE4/4 = 6<br>APOE4/4+HFD = 6	Two-way ANOVA	Total, Genotype, $F (1, 20) = 103.592$, $p < 0.001$, Treatment, $F (1, 20) = 17.001$, $p < 0.001$, Genotype × Treatment, $F (1, 20) = 12$, $p = 0.002$<br>Free, Genotype, $F (1, 20) = 129.911$, $p < 0.001$, Treatment, $F (1, 20) = 26.848$, $p < 0.001$, Genotype × Treatment, $F (1, 20) = 16.875$, $p = 0.001$
S4B	APOE3/3 = 6<br>APOE3/3+HFD = 6<br>APOE4/4 = 6<br>APOE4/4+HFD = 6	Two-way ANOVA	Vessel diameter, Genotype, $F (1, 20) = 281.262$, $p < 0.001$, Treatment, $F (1, 20) = 23.026$, $p < 0.001$, Genotype × Treatment, $F (1, 20) = 26.459$, $p < 0.001$<br>TJ numer, Genotype, $F (1, 20) = 192.794$, $p < 0.001$, Treatment, $F (1, 20) = 11.818$, $p = 0.003$, Genotype × Treatment, $F (1, 20) = 6.787$, $p = 0.017$.
S5A	APOE3/3 = 6<br>APOE3/3+HFD = 6<br>APOE4/4 = 6<br>APOE4/4+HFD = 6	Two-way ANOVA	PPP2CA, genotype, $F (1, 21) = 0.001$, $p = 0.974$, treatment, $F (1, 21) = 0.298$, $p = 0.591$, phenotype × genotype, $F (1, 21) = 0.123$, $p = 0.730$. n = 6 per group<br>PPP2CB, genotype, $F (1, 21) = 2.53$, $p = 0.127$, treatment, $F (1, 21) = 0.193$, $p = 0.665$, phenotype × genotype, $F (1, 21) = 0.001$, $p = 0.974$
S5B	APOE3/3 = 6<br>APOE3/3+HFD = 6<br>APOE4/4 = 6	Two-way ANOVA	PPP2R2C, genotype, $F (1, 21) = 0.004$, $p = 0.949$, treatment, $F (1, 21) = 1.884$, $p = 0.185$, phenotype × genotype, $F (1, 21) = 0.66$, $p = 0.426$

APOE4/4+HFD = 6	PPP2R3A, genotype, $F (1, 21) = 2.53$, $p = 0.127$, treatment, $F (1, 21) = 0.265$, $p = 0.612$, phenotype × genotype, $F (1, 21) = 0.376$, $p = 0.547$<br>PPP2R5B, genotype, $F (1, 21) = 1.48$, $p = 0.238$, treatment, $F (1, 21) = 0.001$, $p = 0.98$, phenotype × genotype, $F (1, 21) = 0.043$, $p = 0.838$<br>PPP2R5D, genotype, $F (1, 21) = 3.435$, $p = 0.0797$, treatment, $F (1, 21) = 0.089$, $p = 0.769$, phenotype × genotype, $F (1, 21) = 0.013$, $p = 0.912$

Uncropped blots

Figure 1F

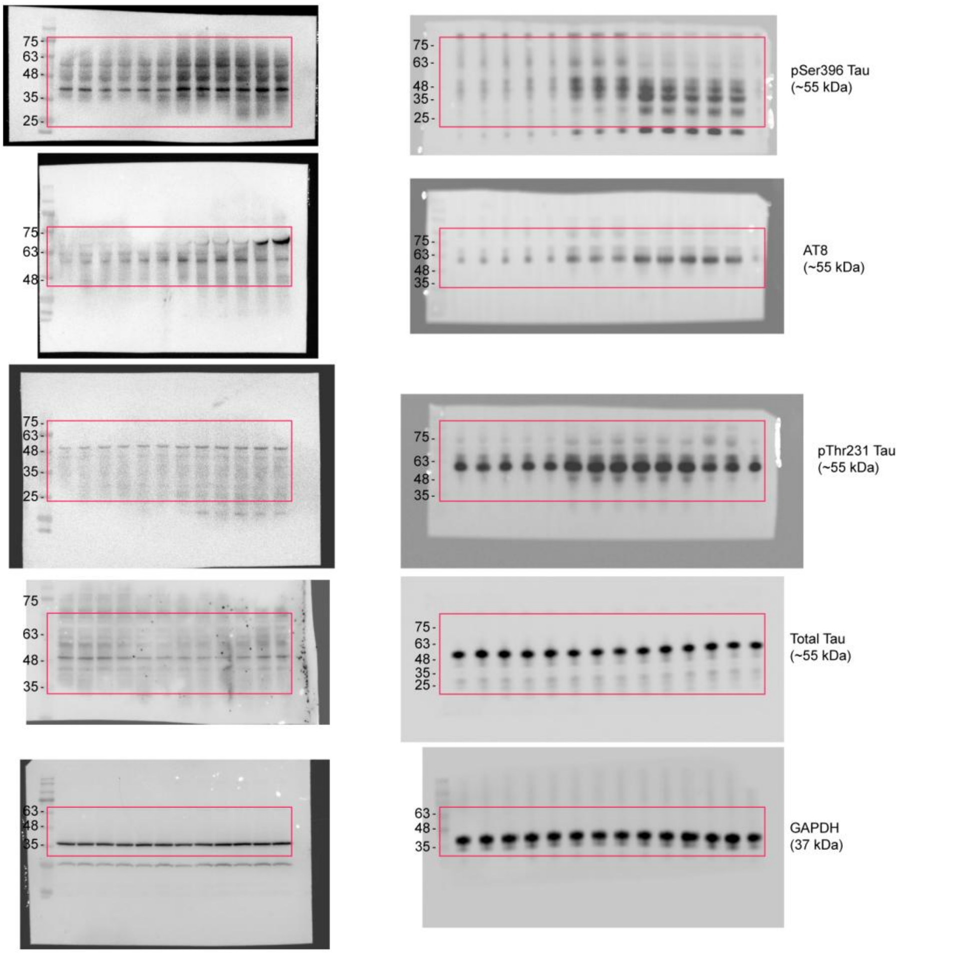

Figure 1N

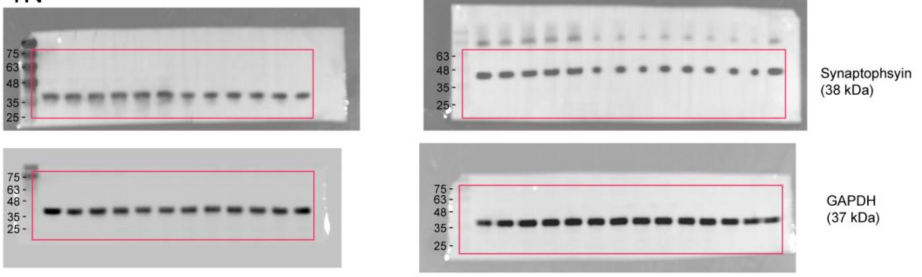

Figure 2A

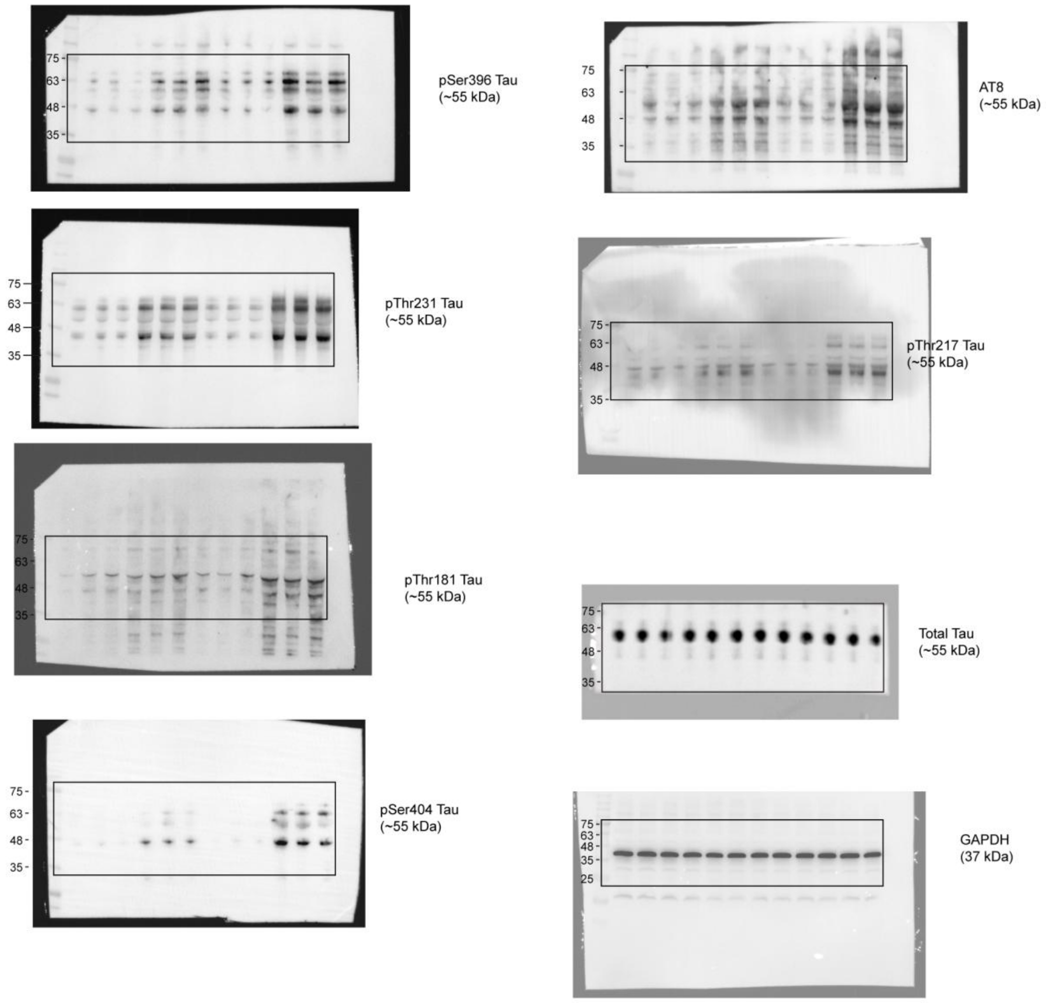
pSer396 Tau
(~55 kDa)
AT8
(~55 kDa)
pThr231 Tau
(~55 kDa)
pThr217 Tau
(~55 kDa)
pThr181 Tau
(~55 kDa)
Total Tau
(~55 kDa)
pSer404 Tau
(~55 kDa)
GAPDH
(37 kDa)

Figure 3A

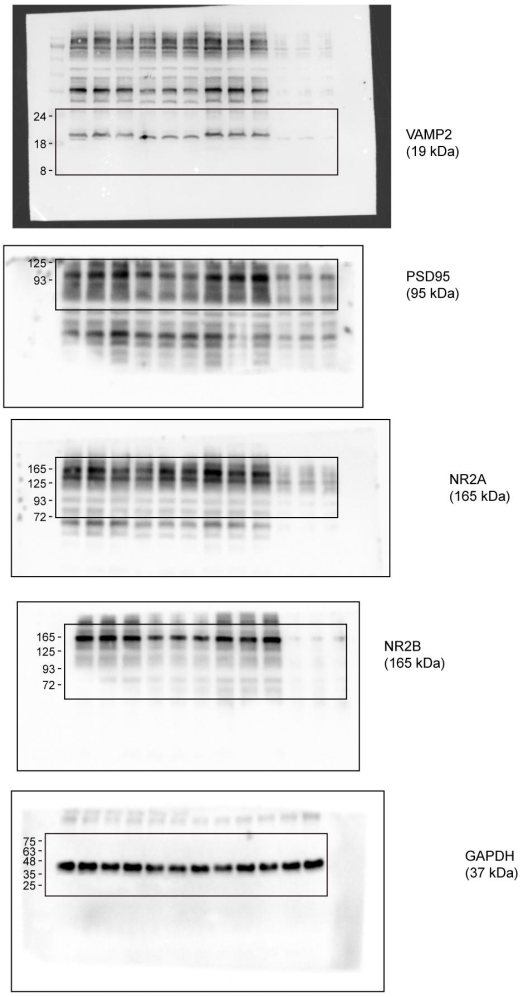
24
18
8
VAMP2
(19 kDa)
125
93
PSD95
(95 kDa)
165
125
93
72
NR2A
(165 kDa)
165
125
93
72
NR2B
(165 kDa)
75
63
48
35
25
GAPDH
(37 kDa)

Figure 4C

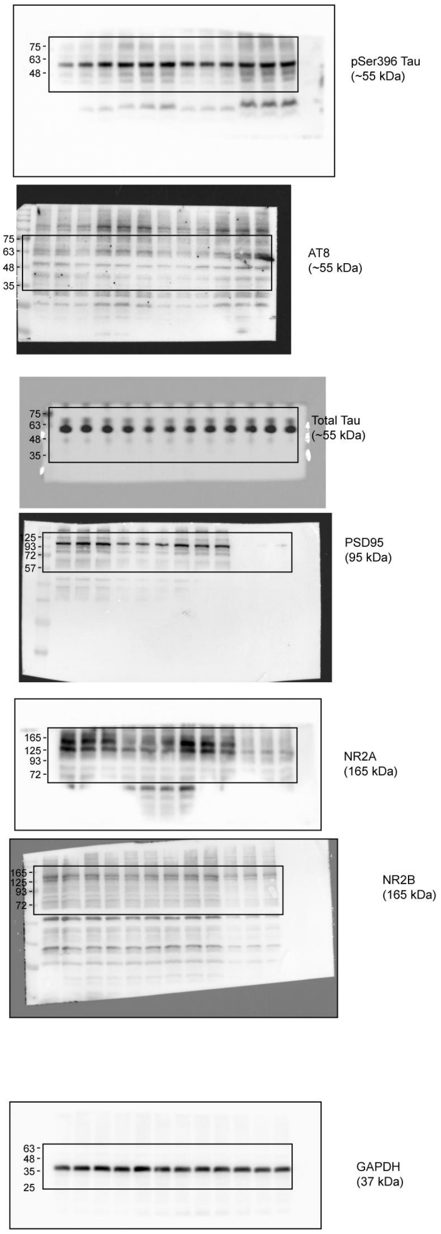
75
63
48
pSer396 Tau
(~55 kDa)
75
63
48
35
AT8
(~55 kDa)
75
63
48
35
Total Tau
(~55 kDa)
125
93
72
57
PSD95
(95 kDa)
165
125
93
72
NR2A
(165 kDa)
165
125
93
72
NR2B
(165 kDa)
63
48
35
25
GAPDH
(37 kDa)

Figure 5

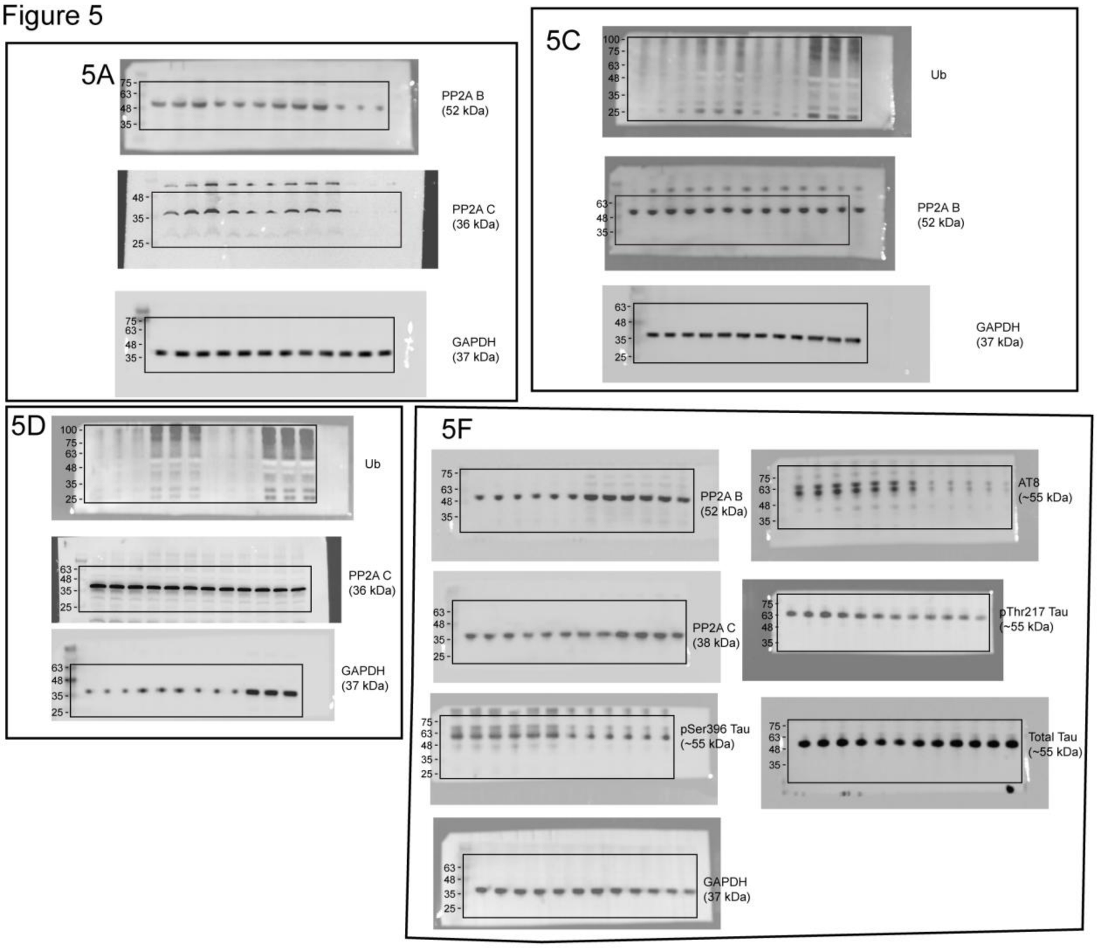
5A
PP2A B (52 kDa)
PP2A C (36 kDa)
GAPDH (37 kDa)
5C
Ub
PP2A B (52 kDa)
GAPDH (37 kDa)
5D
Ub
PP2A C (36 kDa)
GAPDH (37 kDa)
5F
PP2A B (52 kDa)
AT8 (~55 kDa)
PP2A C (38 kDa)
pThr217 Tau (~55 kDa)
pSer396 Tau (~55 kDa)
Total Tau (~55 kDa)
GAPDH (37 kDa)

Figure 5I

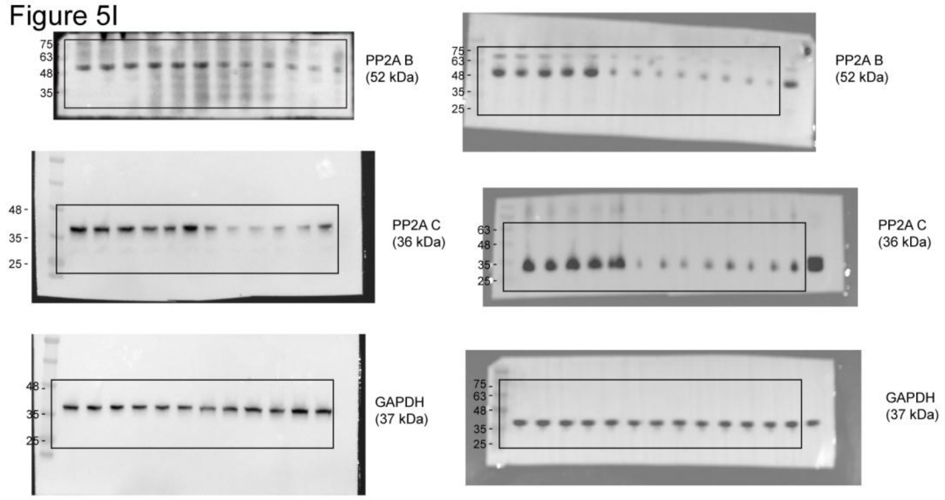

Figure 5L

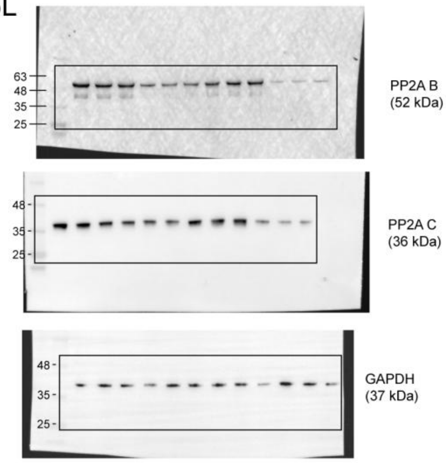

Figure 6I

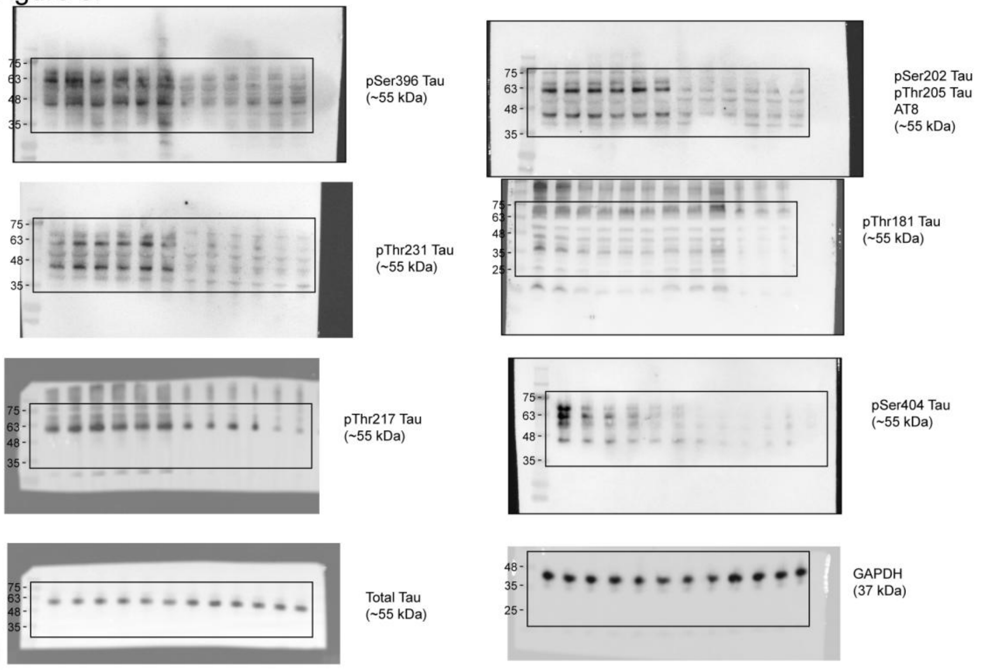

Figure 6K

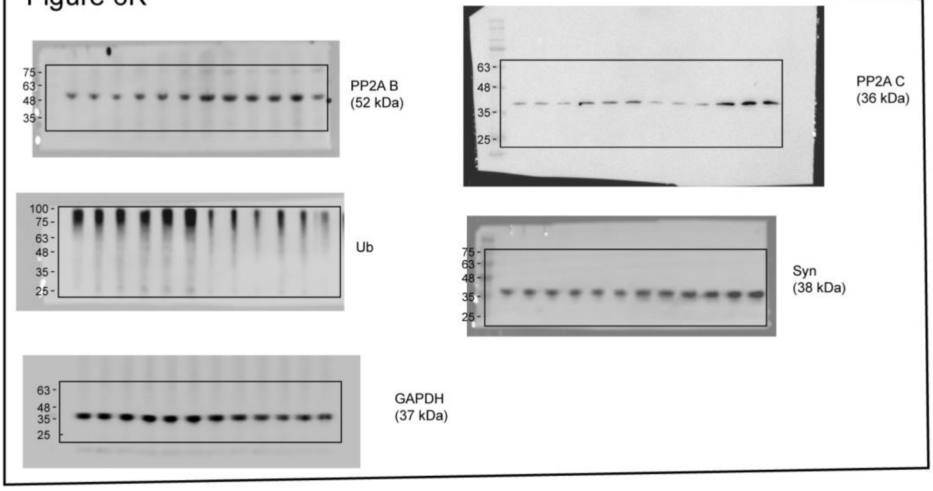

Supplement: Supplementary file 1 — Data S1 [file CNS-31-e70536-s001.pdf]
